# Overexpression of pathogenic tau in astrocytes causes a reduction in AQP4 and GLT1, an immunosuppressed phenotype and unique transcriptional responses to repetitive mild TBI without appreciable changes in tauopathy

**DOI:** 10.1186/s12974-024-03117-4

**Published:** 2024-05-15

**Authors:** Camila Ortiz, Andrew Pearson, Robyn McCartan, Shawn Roche, Nolan Carothers, Mackenzie Browning, Sylvia Perez, Bin He, Stephen D. Ginsberg, Michael Mullan, Elliott J. Mufson, Fiona Crawford, Joseph Ojo

**Affiliations:** 1 The Roskamp Institute, Sarasota, FL USA; 2 The Open University, Milton Keynes, UK; 3 Barrow Neurological Institute, Phoenix, AZ USA; 4 Center for Dementia Research, Nathan Kline Institute, Orangeburg, NY USA; 5 Departments of Psychiatry, Neuroscience and Physiology, and NYU Neuroscience Institute, New York University Grossman School of Medicine, New York, NY USA; 6 James A. Haley Veterans Hospital, Tampa, FL USA

**Keywords:** Astrocytes, Tau astrogliopathy, Traumatic brain injury, Neuroinflammation, Chronic traumatic encephalopathy

## Abstract

Epidemiological studies have unveiled a robust link between exposure to repetitive mild traumatic brain injury (r-mTBI) and elevated susceptibility to develop neurodegenerative disorders, notably chronic traumatic encephalopathy (CTE). The pathogenic lesion in CTE cases is characterized by the accumulation of hyperphosphorylated tau in neurons around small cerebral blood vessels which can be accompanied by astrocytes that contain phosphorylated tau, the latter termed tau astrogliopathy. However, the contribution of tau astrogliopathy to the pathobiology and functional consequences of r-mTBI/CTE or whether it is merely a consequence of aging remains unclear. We addressed these pivotal questions by utilizing a mouse model harboring tau-bearing astrocytes, GFAP^P301L^ mice, subjected to our r-mTBI paradigm. Despite the fact that r-mTBI did not exacerbate tau astrogliopathy or general tauopathy, it increased phosphorylated tau in the area underneath the impact site. Additionally, gene ontology analysis of tau-bearing astrocytes following r-mTBI revealed profound alterations in key biological processes including immunological and mitochondrial bioenergetics. Moreover, gene array analysis of microdissected astrocytes accrued from stage IV CTE human brains revealed an immunosuppressed astroglial phenotype similar to tau-bearing astrocytes in the GFAP^P301L^ model. Additionally, hippocampal reduction of proteins involved in water transport (AQP4) and glutamate homeostasis (GLT1) was found in the mouse model of tau astrogliopathy. Collectively, these findings reveal the importance of understanding tau astrogliopathy and its role in astroglial pathobiology under normal circumstances and following r-mTBI. The identified mechanisms using this GFAP^P301L^ model may suggest targets for therapeutic interventions in r-mTBI pathogenesis in the context of CTE.

**Supplementary Information:**

The online version contains supplementary material available at 10.1186/s12974-024-03117-4.

## Background

Repetitive mild traumatic brain injury (r-mTBI) is associated with an increased risk of neurodegenerative diseases such as Chronic Traumatic Encephalopathy (CTE). Pathognomonic lesions in CTE are characterized by the accumulation of phosphorylated tau in neurons, with or without tau-bearing astrocytes (i.e., 4R-tau astrogliopathy) within the depths of cortical sulci [1]. Tau astrogliopathy may play a key role in dysfunction of the central nervous system (CNS) homeostasis, blood–brain barrier (BBB) integrity, and trophic and metabolic support for neurons. In vitro studies have indicated that overexpression of 4R-tau in astrocytes renders them more vulnerable to oxidative stress and with an inability to clear synaptic glutamate, which increases the vulnerability of neurons to hyperexcitability and death [2, 3]. Transgenic mouse models of astroglial tauopathy manifest a significant reduction of glial glutamate transporters, GLAST and GLT1, as early as 5 months, in regions where astrocytic tau accumulation is most robust (e.g., brainstem and spinal cord) [4, 5].

To date, the effect of r-mTBI-induced tau astrogliopathy upon astroglial homeostasis and pathobiology remains to be determined. The present study interrogated the histopathological, biochemical and transcriptional effects of tau astrogliopathy on astroglial pathobiology under normal circumstances and after r-mTBI. Additionally, we were also interested in comparing r-mTBI-induced astroglial responses to endogenous overexpression of mutant tau (GFAP^P301L^ model) versus exogenous neuronal tau overproduction. We, therefore, used a mouse model of neuronal tauopathy, the CaMKIIα^P301L^ model, also referred to as rTg4510 mice. We also assessed non-TBI related pathological effects of astroglial tauopathy by using the r-sham groups (i.e., GFAP^P301L^ versus WT and CaMKIIα^P301L^ versus WT). Finally, we compared the genetic expression of tau-bearing astrocytes in the transgenic GFAP tau mouse to astrocytes extracted from human autopsy cases of CTE.

We hypothesized that overexpression of human pathogenic tau leads to an increased astroglial and microglial response, dysregulation of astroglial homeostatic markers and transcriptional dysregulation compared to tau^WT^ astrocytes (i.e., astrocytes from WT and CaMKIIα^P301L^ mice). In addition, we posit early exposure to r-mTBI (pre-onset of tau astrogliopathy) will exacerbate tau accumulation in astrocytes and brain-wide tauopathy, increase astroglial and microglial responses, cause more pronounced dysregulation of astroglial homeostatic markers and transcriptional changes in mice harboring tau-bearing astrocytes compared to WT and CaMKIIα^P301L^ models. We further expect that tau-bearing astrocytes manifest changes in astroglial biology faithfully recapitulating pathological changes of astrocytes extracted from postmortem CTE cases.

## Methods and materials

### Animals

**Fig. 1:**
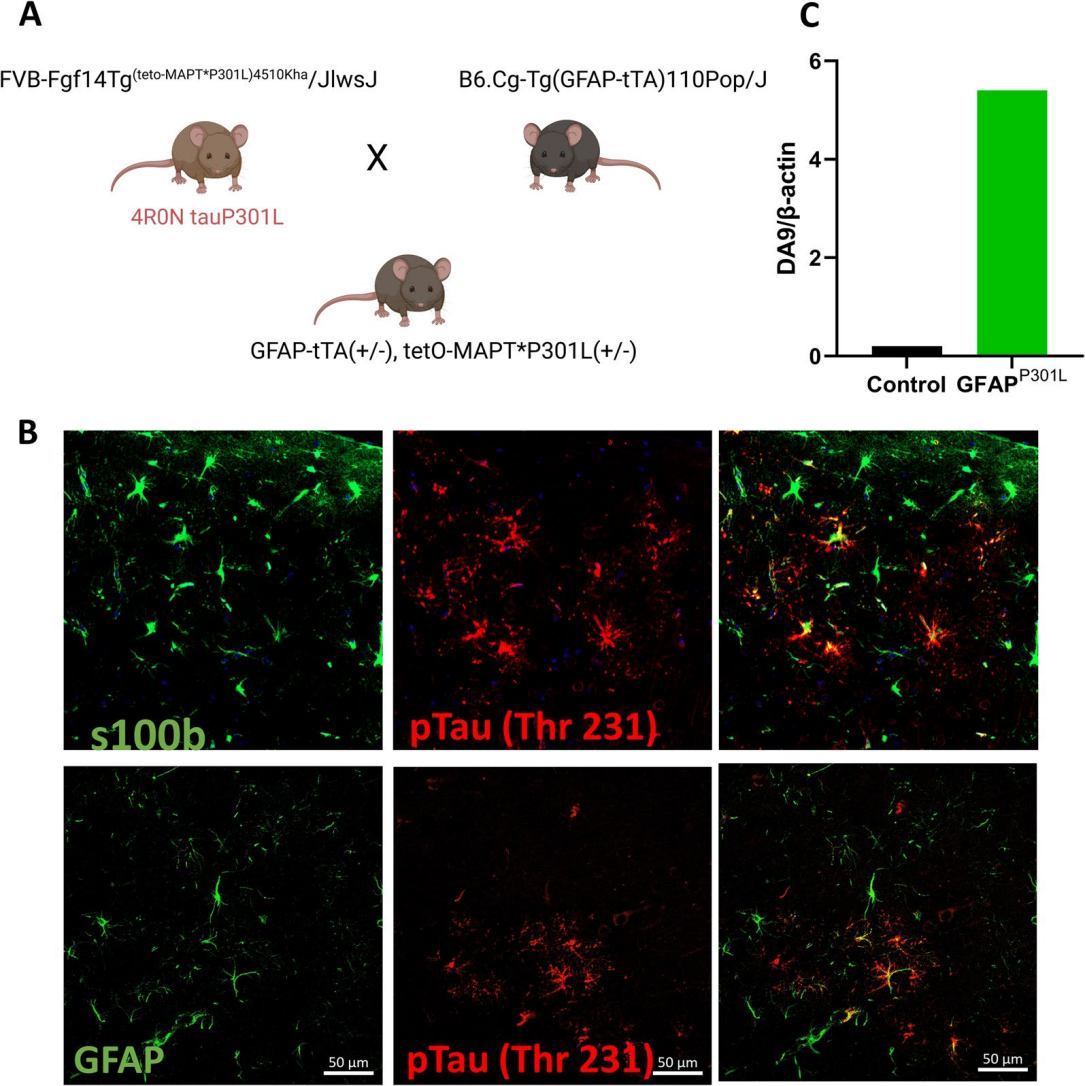
Development of an inducible/reversible conditional mouse model of tau astrogliopathy. **A** Schematic drawing depicting the generation of GFAP-tTA(±)/tet0-MAPT*P301L(±) mice (referred to as GFAP^P301L^ mice) by crossing tetO-MAPT*P301L transgenic mice (i.e., FVB-Fgf14Tg(tet0-MAPT*P301L)4510kha/JlwsJ) with B6.Cg-Tg(GFAP-tTA)/110Pop/J mice that express a tetracycline-controlled transactivator protein (tTA) driven by the human glial fibrillary acidic protein (*GFAP*) promoter. This bitransgenic mouse allows Tet-Off/Tet-On expression of a P301L mutant variant of human four-repeat microtubule-associated protein tau (4R0N tauP301L) under control of GFAP promoter, specifically in astrocytes. **B** Qualitative micrographs of astrocyte colocalization with phosphorylated tau using S100 $\beta$/pTau T231 immunofluorescence (upper panel) and GFAP/pTau T231 (lower panel). **C** Quantitative immunoblot of total tau protein (DA9) levels in astrocytes of GFAP-P301L vs non-carrier (control) mice. DA9 was normalized to house-keeper—$\beta$ actin

This study investigated three-month-old male and female C57BL/6 mice (n = 12), GFAP^P301L^ (n = 12), and CaMKIIα^P301L^ (n = 12). Because GFAP^P301L^ mice were no longer available from Dr. Trojanowski [4, 5], we generated a GFAP^P301L^ transgenic mouse (Fig. 1A–C) by crossing GFAP-tTa mice (a generous gift from Dr. Brian Popko, Northwestern University, IL) with tetO-MAPT*P301L (cat#015815; The Jackson Laboratory, ME, USA)*.* We favored the utilization of human P301L mutant tau over human WT tau due to its preferential aggregation of 4R tau [6] which is particularly relevant in various primary tauopathies such as CTE, progressive supranuclear palsy (PSP), corticobasal degeneration (CBD), and age-related tau astrogliopathy (ARTAG) [7]. GFAP^P301L^ and CaMKIIα^P301L^ (Tg4510) were genetically engineered to express the P301L MAPT mutation (4R/0N-human) under the GFAP or CamMKIα promoter leading to tau expression in astrocytes or neurons, respectively. Both genetically engineered models were on an FVB-C57BL/6 background. Mice were housed in a 12 h light/dark cycle with food and water ad libitum*.* All experiments were performed in accordance with Office of Laboratory Welfare and National Institutes of Health guidelines with Roskamp Institute Institutional Animal Care and Use Committee approval.

### Traumatic brain injury administration

**Fig. 2:**
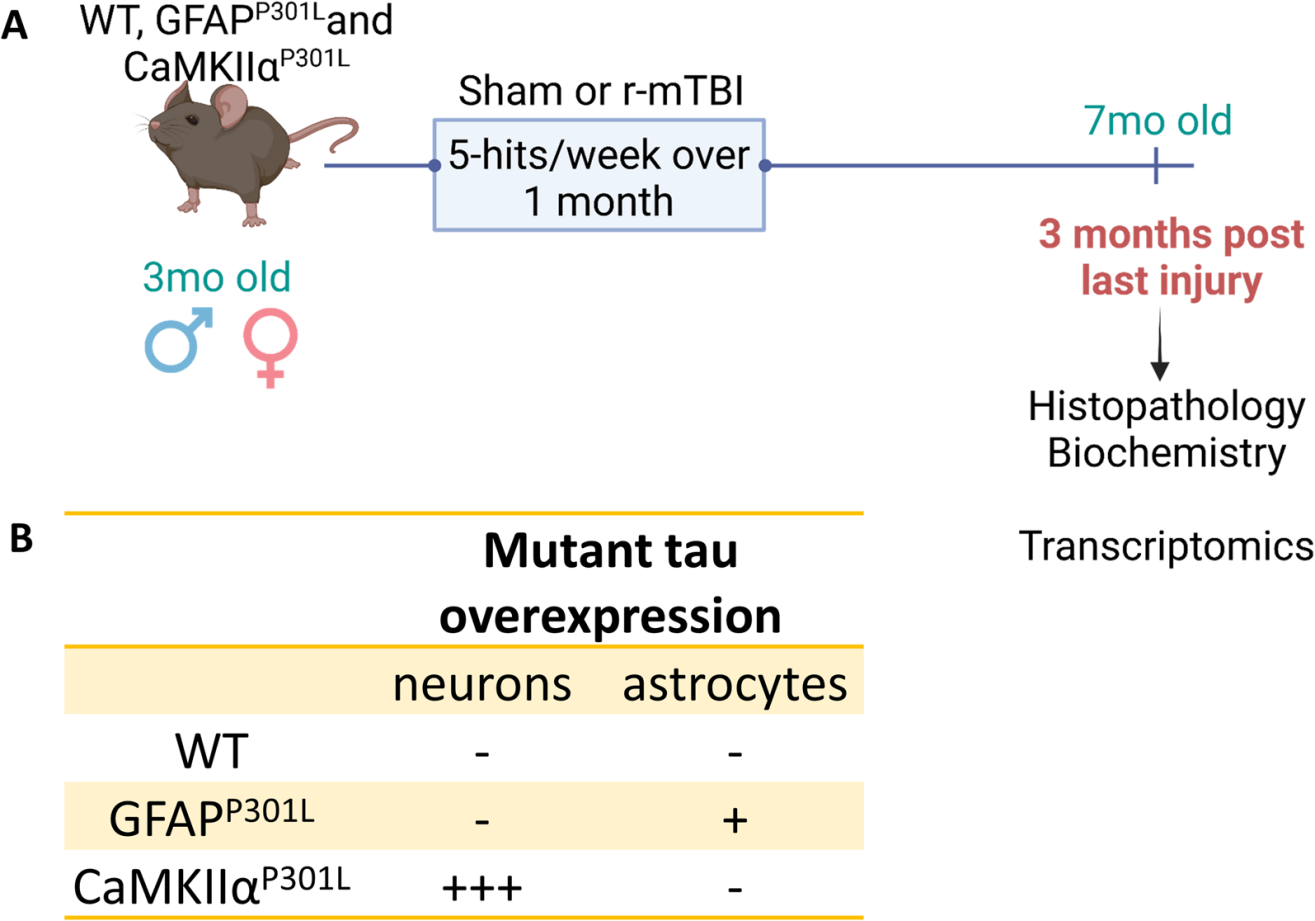
Timeline of experiments and features of mouse models. **A** Three-month-old wild-type (WT), GFAP^P301L^, and CamkIIα^P301L^ were subjected to mild TBI every weekday for 5 days a week for four weeks resulting in 20 hits in a month. Three months post-last injury brain mouse tissue was collected for histopathological, biochemical and transcriptional analyses. **B** mutant tau expression in the three mouse models utilized for this study. − none; + some; +  +  + abundant

Our well-characterized closed-head mild TBI [8–11] was used in a chronic repetitive injury paradigm involving 20 mTBI over the course of four weeks, as described previously [12]. Briefly, mice subjected to r-mTBI were anesthetized with 1.5 L/min and 3% isoflurane for 3 min and the injury site was shaved. Mice were then positioned in a stereotaxic frame (Stereotaxic Instrument, Stoelting, Wood Dale, Illinois) attached to an impactor (Impact One Stereotaxic Motorized Impactor, Richmond, Illinois). Mice were placed on a heating pad to maintain body temperature at 37 °C during head impacts, which were administered to the area of the shaved head above the mid-sagittal suture in the central area of the skull. Head impact was performed using a 5 mm blunt metal impactor tip with 1mm strike depth. The impact was delivered at 5 m/s with a force of 72N. To prevent hypothermia post-injury mice were placed on a heating pad set at 37 °C until they gained consciousness. Mice were injured every weekday for 5 days a week for 4 weeks resulting in 20 hits in a month. Sham counterparts were exposed to isoflurane with the same frequency and duration. Mice were sacrificed 3 months post-last injury for histopathological, biochemical, or transcriptomic analyses (Fig. 2).

### Intracerebral injection of AAV vector

Ten-month-old TauKI naïve mice were anesthetized with 4%isoflurane with 1.5 L/min oxygen and then placed on the stereotaxic platform. A single injection of ketoprofen (5 mg/kg; Patterson Veterinary, USA) was administered subcutaneously before surgery for pre-operative analgesia. The head was shaved, and the exposed area was sterilized using betadine solution 5% povidone-iodine. Using a scalpel, a midline incision was made in the skin to expose the skull. Mice received unilateral intracranial injections into the cortex (AP: − 1; ML: − 1 DV: − 1.3 from Bregma) through a small burr hole in the skull. Four microliters of virus vector (AAV5-GFAP-htau^P301L^-GFP; 3.37 × 10^13^ vg/mL) were injected using a 10 μl Hamilton syringe with a 30G needle and a rate of 0.2 μl/min. The needle was kept in place for 10 min before withdrawal to avoid backflow. The incision was closed using webglue (Pivetal, USA). Mice were monitored until they recovered consciousness, at which point they were administered Ethiqa (3.25 mg/kg; Covetrus, USA) as a post-operative analgesic (with 72 h duration). Mice were daily monitored for 72 h after the injection. Transfection was allowed to occur for 6 weeks after the injection. Mice were then subjected to the 20-hit r-mTBI paradigm described above. Six months after the last injury brains were collected in 4% paraformaldehyde (PFA) in phosphatase-buffered saline (PBS) and stored at 4 °C for 48 h. Brains were transferred to 20% sucrose PBS solution and stored at 4 °C until they were sectioned using a vibratome Leica VT1200S (Leica, USA). Coronal brain section (25 μm) were collected and immunostained with AT8 in well-plates. Subsequently sections were mounted on glass slides and imaged using ZEISS LSM 800 (ZEISS, Germany).

### Immunohistochemistry

Mice were deeply anesthetized with isoflurane and perfused transcardially with 4 °C cold phosphate-buffer saline (PBS), decapitated and the right hemibrain was placed in 4% PFA in PBS solution for 24-48 h, then were dehydrated and paraffin-embedded using the Tissue-Tek^®^ VIP and Tissue-Tek^®^ TEC (Sakura, USA), respectively. Brains were sectioned at 6 μm using a Leica RM2235 microtome (Leica, USA) and mounted on glass slides. Sections were deparaffinized using HistoClear^®^ (National Diagnostics, USA) and rehydrated in a decreasing gradient of ethanol prior to immunohistochemistry.

*Immunofluorescence:* Following rehydration, slides were submerged in PBS for 5 min to wash off the excess ethanol. For better detection of GFAP/RZ3 (Thr 231)/S100β antigen retrieval was performed using an acidic buffer (pH 6). The antigen retrieval solution was warmed using a pressure cooker, the antigen solution was prepared, slides immersed and heated for 7 min in a microwave. Following antigen retrieval, sections were blocked with 5% normal donkey serum (NDS) dissolved in 0.1% TritonX-100 phosphate-buffered saline (PBST) at room temperature (RT) for 1 h. Sections were immunostained using primary antibodies (supplementary Table 1) in a PBST solution containing 1% NDS. After overnight incubation at 4 °C, sections were rinsed with PBS and transferred to a solution containing the appropriate fluorophore-conjugated secondary antibody (Alexa Fluor antibodies 648 nm, 555 nm, 488 nm 1:500, Thermofisher, MA, USA) at RT for 1 h. Autofluorescence was quenched with an autofluorescence removal reagent (Millipore Sigma, MA, USA). Sections were mounted using anti-fade mounting media with DAPI (Abcam, MA, USA). Z-stack images of 6 μm-thick sections were collected using a ZEISS LSM 800 (ZEISS, Germany) confocal microscope.

*Immunohistochemistry:* Following rehydration, slides were submerged in PBS for 5 min to remove excess ethanol. Slides were immersed in hydrogen peroxide for 15 min to deactivate endogenous peroxidases. Antigen retrieval was performed using a basic buffer (pH 8). Sections were incubated with 2.5% normal goat serum (VectorLabs, CA, USA) at RT for 1 h and subsequently incubated with primary antibody at 4 °C overnight. Slides were rinsed in PBS and incubated with horse radish peroxidase (HRP)-labeled secondary antibody (VectorLabs, CA, USA) at RT for 1 h. Immunohistochemical signal was developed using 3,3’-Diaminobenzidine (DAB) (VectorLabs, CA, USA). After light counterstaining with hematoxylin, tissue was dehydrated in an increasing series of ethanol (80%, 95%, and 100%). Next, slides were cover-slipped using toluene (Milipore Sigma, MA, USA). Images were collected using an Olympus DP72 microscope (Olympus, PA, USA).

### Microscopy and image analysis

*Confocal microscopy:* Multiple regions of interest in the cortex beneath the impact area were analyzed for each marker. A minimum of 20 microscopic fields (5 images per sagittal section, 4 sections per mouse brain) were imaged using a 20 × objective lens (total magnification 200×). Images from individual channels (wavelengths 648 nm, 555 nm, 488 nm, and 450 nm) were used independently for subsequent percentage area analysis. The immunoreactivity of cell markers was measured by quantitative image analysis performed blinded by the investigator using ImageJ software. Quantification of GFAP^+ve^/RZ3^+ve^ astrocytes was performed throughout the entire cortex of the 4 sagittal sections.

*Bright-field microscopy:* Multiple regions of interest in the cortex (ventral to the impact site) were analyzed for Iba1. A minimum of 20 microscopic fields (5 images per sagittal section, 4 sections per mouse brain) were imaged using a 40 × objective lens. Iba1 immunoreactivity was measured blinded by the investigator using ImageJ software. Before quantitative analysis, color deconvolution was applied on bright-field images to separate the DAB reaction from the hematoxylin staining. Percentage area analysis was then performed using the “DAB” set of images.

### Western blotting

Hippocampal samples were sonicated in 250 μl of mammalian protein extraction reagent, M-PER, (plus protease and phosphatase inhibitors, Thermofisher, MA, USA) to ensure maximal protein extraction. Samples were centrifuged at 14,000 rpm for 20 min at 4 °C to pellet debris. Total amount of protein was determined using the Bicinchoninic acid (BCA) assay (Thermofisher, MA, USA). A total of 30 μg of tissue homogenate were mixed with a denaturing buffer containing 10% β-mercaptoethanol and boiled at 95 °C for 10 min. Samples were then loaded on 4–15% SDS-PAGE gels for protein separation and then transferred onto PVDF membranes overnight at 4 °C. Transferred membranes were blocked with 5% non-fat milk in 0.05% Tween 20 Tris-buffered Saline (TBST) for 1 h at RT. Membranes were incubated with the primary antibodies (see Suppl. Table 1) overnight at 4 °C. Membranes were washed in TBST 3 times (5 min each) prior to exposure of HRP-conjugated secondary antibodies (Cell Signaling, MA, USA) for 1 h at RT. Membranes were washed in TBST and deionized water and developed by chemiluminescent (ECL or FEMTO) (Thermofisher, MA, USA). Imaging was performed using a ChemoDoc MP imager (BioRad, CA, USA) and densitometry analysis was performed with Image Lab software (Version 6.1, BioRad, CA, USA). Because standardized housekeeper proteins (e.g., β-actin and GAPDH) displayed genotype-dependent variability, total protein obtained by Ponceau-S (Millipore, MA, USA) staining was used to normalize the data.

### Statistical analyses for histopathological and biochemical analyses

Statistical analyses were performed using GraphPad Prism (Version 9, Dotmatics, CA, USA). Data were tested for normality using the Shapiro–Wilk test. Statistical analysis was obtained using either t-test or two-way ANOVA accompanied by the Benjamini, Krieger, and Yekuteli test to correct multiple comparisons. When datasets were not normally distributed, the datasets were log-transformed. If after transformation, datasets maintained a non-normal distribution, the Mann–Whitney test or Kruskal–Wallis non-parametric test were performed. Data are presented as mean ± SEM. A p value (p < 0.05) was considered statistically significant following post-hoc testing. Asterisks in the Results represent different p value: *p < 0.05; **p < 0.01, and ***p < 0.001.

### Human brain tissue

Hippocampi of 11 male athletes who participated in American football with a history of repetitive mild TBI were provided to an author (EJM) by Boston University School of Medicine [13, 14] (see Table 1). The human CTE cases (n = 11 with a mean age of 84.3) were diagnosed as stage-IV, the most severe category, according to the neuropathological McKee and colleagues’ criteria for this disorder [15]. In addition, we evaluated nine elderly age-matched controls (2 males and 7 females) with a mean age of 77.3 (see Table 1) lacking a history of brain trauma.

**Table 1:** Demographics of human CTE and healthy control (HC) cases used for microdissection and gene array analysis of hippocampal astrocytes

	HC	CTE IV
N	9	11
Sex (M/F)	2/7	11/0
Race (C/AA)	0/9	9/2
Sport Played (Soc/Rug/Hoc/Foot)	N/A	0/0/0/11
Sport Level (am/co/semipro/pro)	N/A	0/3/0/8
Military Service (Y/N)	N/A	2/9
Age begun	N/A	11.3 (±2.24)
Years of play	N/A	11.3 (±2.24)
Age of symptom onset	N/A	59.9 (±14.58)
Years retirement to symptom onset	N/A	31.9 (±16.28)
Years symptom onset to death	N/A	17.4 (±10.96)
Age at death	84.3 (±12.25)	77.3 (±8.26)

*HC* healthy controls, *M* male, *F* female, *C* Caucasian, *AA* Afro-American, *Soc* soccer, *Rug* rugby, *Hoc* hockey, *Foot* American football, *am* amateur, *co* college, *semipro* semi-professional, *pro* professional, *Y* yes, *N* no, *N/A* not applicable

### Immunofluorescence of human tissue

Double stain was used to evaluate whether astrocytes display tau pathology using antibodies to the astrocytic marker GFAP and phosphorylated tau at S396/404, PHF-1, in the hippocampus of CTE stage IV cases [16]. One section per case was incubated overnight at room temperature with both GFAP (1:1000, DAKO Denmark) and PHF-1 (1:1000, gift from Peter Davies) after 10 min citric acid pH 6 antigen retrieval. Then, the appropriate secondary antibodies were applied for 1 h at room temperature as followed: first Cy5-conjugated donkey anti-mouse IgG for PHF-1 (1:400, Jackson Immuno-research, West Grove, PA) and secondly, after several washes, Cy2-donkey anti-rabbit IgG for GFAP (1:400, Jackson Immuno-research). DAPI, a nuclear fluorescence stain, was employed at 1:2000 at room temperature for 10 min. Auto-fluorescence was blocked with Auto-fluorescence Eliminator Reagent (Millipore, MA, USA) according to manufacturer’s instructions and cover-slipped with aqueous mounting media (Thermo Scientific). Dual immunofluorescence was visualized with the aid of a Revolve Fluorescent Microscope (Echo laboratories, CA, USA) with excitation filters for DAPI (pseudocolored green), Cy2 (emission green; pseudocolored red) and Cy5 (pseudocolored blue) as described previously [17].

### Astroglial isolation

Following transcardial perfusion, left hemibrains were removed and placed into a petri dish on wet ice. Enzymatic tissue digestion was performed using the Adult Brain Dissociation Kit (Miltenyi Biotec, CA, USA). Brains were minced using a sterile scalpel blade and transferred to a 15 mL conical tube containing 1950 μl of enzyme mix 1 (enzyme P and buffer Z) and 30 μl of enzyme mix 2 (enzyme A and buffer Y), incubated in the enzyme mix for 30 min and then further dissociated using repeated pipetting. Samples were briefly centrifuged and filtered through a 70 µm cell strainer to achieve a single-cell suspension. Single cells were resuspended in 900 µl of Debris removal solution mixed with 3.1 mL of PBS containing 0.5% fetal bovine serum (FBS) (PBS buffer) and then transferred to a fresh 15 mL falcon tube, overlayed with 4 mL of PBS buffer and centrifuged at 3000×*g* for 10 min. Cells were rinsed in PBS to remove any remaining debris and centrifuged for 10 min. The supernatant was aspirated, and cells were labeled with FcR blocking Reagent (Miltenyi Biotec, CA, USA) for 10 min at 4 °C to avoid non-specific binding of the astrocyte-specific magnetic bead-conjugated ASCA2 antibody (Miltenyi Biotec, CA, USA). Samples were subsequently incubated with ASCA2 antibody for 15 min at 4 °C, then loaded onto a pre-conditioned LS separation column and rinsed three times to remove unlabeled cells. To elute ACSA2^+ve^ cells, the LS column was removed from the magnet and PBS was used to elute the sample. Enriched ACSA2^+ve^ cells were centrifuged at 1000*g* for 5 min and stored at − 80 °C in Trizol until RNA extraction. Two left hemibrains from two different mice that belonged to the same group were combined to collect enough material for one RNA sample. In total we had an n = 3 RNA samples per group per genotype for this analysis. These hemibrains were depleted of hippocampi.

### RNA isolation and sequencing

*RNA isolation*: RNA was extracted from astrocytes using a Trizol-based protocol (Chomczynski and Mackey, 1995). Briefly, 500 µl of Trizol was added to cell isolates obtained from the astrocyte isolation procedure. Samples were sonicated and allowed to dissociate at RT for 5 min. Samples were centrifugated at 14000rpm for 15 min. Post-centrifugation, three layers were formed: the top-most layer was the aqueous phase and the one containing RNA. The aqueous layer was transferred to a centrifuge tube for further processing. Additionally, 1 µl of glycogen, per sample, was added to enhance RNA extraction. Serial centrifugation steps accompanied by ethanol washes yielded the RNA pellet that was used for RNA sequencing.

*RNA sequencing:* RNA samples were sent to GENEWIZ LLC (South Plainfield, NJ, USA) for integrity checks (average RIN values = 5), library preparation and astrocyte-specific bulk sequencing. RNA sequencing libraries were prepared from 100 ng of RNA using NEB Next Ultra RNA Library Prep Kit for Illumina. Messenger RNA was first enriched with Oligod(T) beads and fragmented, with the 1st and 2nd strand cDNA subsequently synthesized. cDNA fragments were end-repaired and adenylated, and universal adaptor ligated to fragments, followed by index addition and library enrichment with limited cycle PCR. The sequencing library was validated on the Agilent TapeStation and quantified using Qubit 20 Fluometer as well as qPCR. The sequencing libraries were clustered on one lane of a flowcell. After clustering, the flowcell was loaded on the Illumina HiSeq4000 according to the manufacturer’s instructions. The samples were sequenced using a 2 × 150 Paired End configuration. Raw sequence data from Illumina HiSeq was converted into Fastq files and de-multiplexed.

### Bioinformatic analyses of mouse astrocytes

Bioinformatic analyses were obtained using the public platform usegalaxy.org. RNA sequences received from GENEWIZ are uploaded to the platform where raw data undergoes a quality check (FastQC on individual files followed by MultiQC to obtain a quality report of all files), and then the sequence reads are trimmed to remove adapter sequences, poly-N-containing reads and low quality reads (Phred score <25) (TRimmomatic followed by MultiQC), and mapped to the Mus musculus GRCm38 reference genome (HISAT2). Output files were subjected to labeling of duplicated molecules (MarkDuplicates) for its posterior removal when measuring the gene expression in FPKM (fragments per kilobase million) (featureCounts). Data was inspected for outlying samples using unsupervised hierarchical clustering and principal component analysis. Combat batch correction was applied to combine the datasets and reduce systematic sources of variability. Differential gene expression analysis was conducted to determine relationships between gene expression levels in the injury group versus their sham counterparts (DESeq2). The IDs of the genes were obtained using annotateMyIDs. The covariates were included in all models to adjust for any potential confounding influence on gene expression between main group effects. This was conducted using the Wald test (in DESeq2Genes with FDR adj p < 0.05 classified as differentially expressed genes (DEG). Values with an adjusted -p value of < 0.05 were processed for pathway enrichment analysis using Ingenuity Pathway Analysis (IPA, QIAGEN). In rare instances where DEGs exceeded 5000 in number, we added another cut-off parameter for the expression log2 fold change ± 1.2.

### Single population gene expression analysis on human hippocampal CTE astrocytes

Single GFAP-immunolabeled human hippocampal astrocytes (a total of 50–100 astrocytes per CTE case pooled/per assay) from CTE Stage IV cases (*n* = 11) were microaspirated by laser capture microdissection (PALM MicroBeam C IP, Carl Zeiss MicroImaging Inc., Thornwood, NY) [13, 18].

Microdissected astrocytes were homogenized in Trizol solution and RNAs were extracted for reverse transcription in the presence of the poly d (T) primer (100 ng/mL) and TC primer (100 ng/mL) in 1 × first strand buffer, 500 μM deoxyribonucleotide triphosphate (dNTP)s, 5 mM dithiothreitol (DTT), 20 U of SuperRNase Inhibitor, and 200 U of reverse transcriptase. Single-stranded complementary DNAs (cDNAs) were digested with RNase H and re-annealed with the primers in a thermal cycler: RNase H digestion step at 37 °C, 30 min; denaturation step at 95°C, 3 min; and primer reannealing step at 60 °C, 5 min. This step generated cDNAs with double-stranded regions at the primer interface [19–22]. Samples were then purified by column filtration (Montage PCR filters; Millipore, MA, USA). RNAs hybridization probes were synthesized by in vitro transcription with the use of P incorporation in 40 mmol/L Tris (pH 7.5); 6 mmol/L MgCl2; 10 mmol/L NaCl; 2 mmol/L spermidine; 2.5 mmol/L DTT; 125 μmol/L adenosine triphosphate (ATP), guanosine triphosphate (GTP), and cytidine triphosphate (CTP); 2.5 μmol/L cold uridine triphosphate (UTP); 20 U of RNase inhibitor; 2 kU of T7 RNA polymerase (Epicentre, Madison, WI); and 60 μCi of 33P-UTP (PerkinElmer, Waltham, MA). The labeling reaction was performed at 37 °C for 4 h. Radiolabeled terminal continuation RNA probes were hybridized to custom-designed microarrays without further purification [19–22]. Gene array expression was run in triplicate for each case.

*Custom-designed microarray platforms and data analysis:* Platforms consist of 1 μg of linearized cDNA purified from plasmid preparations adhered to high-density nitrocellulose (Hybond XL, GE Healthcare, Piscataway, NJ). cDNAs were verified by sequence analysis and restriction digestion. Approximately 864 cDNAs were used on our custom array platform [13, 18]. Arrays were hybridized for 24 h in a solution consisting of 6 × saline-sodium phosphate-ethylenediaminetetraacetic acid, 5 × Denhardt’s solution, 50% formamide, 0.1% sodium dodecyl sulfate (SDS), and denatured salmon sperm DNA (200 μg/mL) at 42°C in a rotisserie oven [19]. Following the hybridization protocol, arrays were washed sequentially in 2 × saline sodium citrate (SSC)/0.1% SDS, 1 × SSC/0.1% SDS, and 0.5 × SSC/0.1% SDS for 15 min each at 37 °C. Arrays were placed in a phosphor screen for 24 h and developed on a phosphor imager (Storm 840, GE Healthcare, Piscataway, NJ).

Hybridization signal intensity was determined utilizing ImageQuant software (GE Healthcare). Briefly, each array was compared with negative control arrays utilizing the respective protocols without input RNA [19–21]. Expression of TC amplified RNA bound to each target minus background was expressed as a ratio of the total hybridization signal intensity of the array (a global normalization approach) [19–21]. Global normalization effectively minimizes variation because of differences in the specific activity of the synthesized probe and the absolute quantity of the probe. Data analyzed in this manner does not allow the absolute quantification of mRNA levels generated [19]. However, an expression profile of relative changes in mRNA levels was generated. Relative changes in total hybridization signal intensity and individual mRNAs were analyzed by one-way ANOVA with post hoc Neumann-Keuls test analysis [19–21]. The level of significance was set at p < 0.01 for individual comparisons.

## Results

Three-month-old WT, GFAP^P301L^, and CaMKIIα^P301L^ mice were subjected to our 20-hit model of r-mTBI (or r-sham), and 3 months after the last injury (at the age of 7 months), we performed histopathological and biochemical analyses on brain tissue, and transcriptomic analyses on isolated ex-vivo primary astrocytes to investigate the effects of tau astrogliopathy on brain levels of phosphorylated and total, neuroinflammation (astrocyte and microglial reactivity), astrocytic homeostasis and astrocyte-specific pathological response under normal (no TBI) circumstances and three months post-last injury. For the present study, normal circumstances refer to data from the sham cohorts of each genotype at 3 months post-last isoflurane exposure.

### Effects of astrocytic versus neuronal tau on tau astrogliopathy and brain levels of phosphorylated and total tau in the cortex and hippocampus under normal circumstances and following r-mTBI

**Fig. 3:**
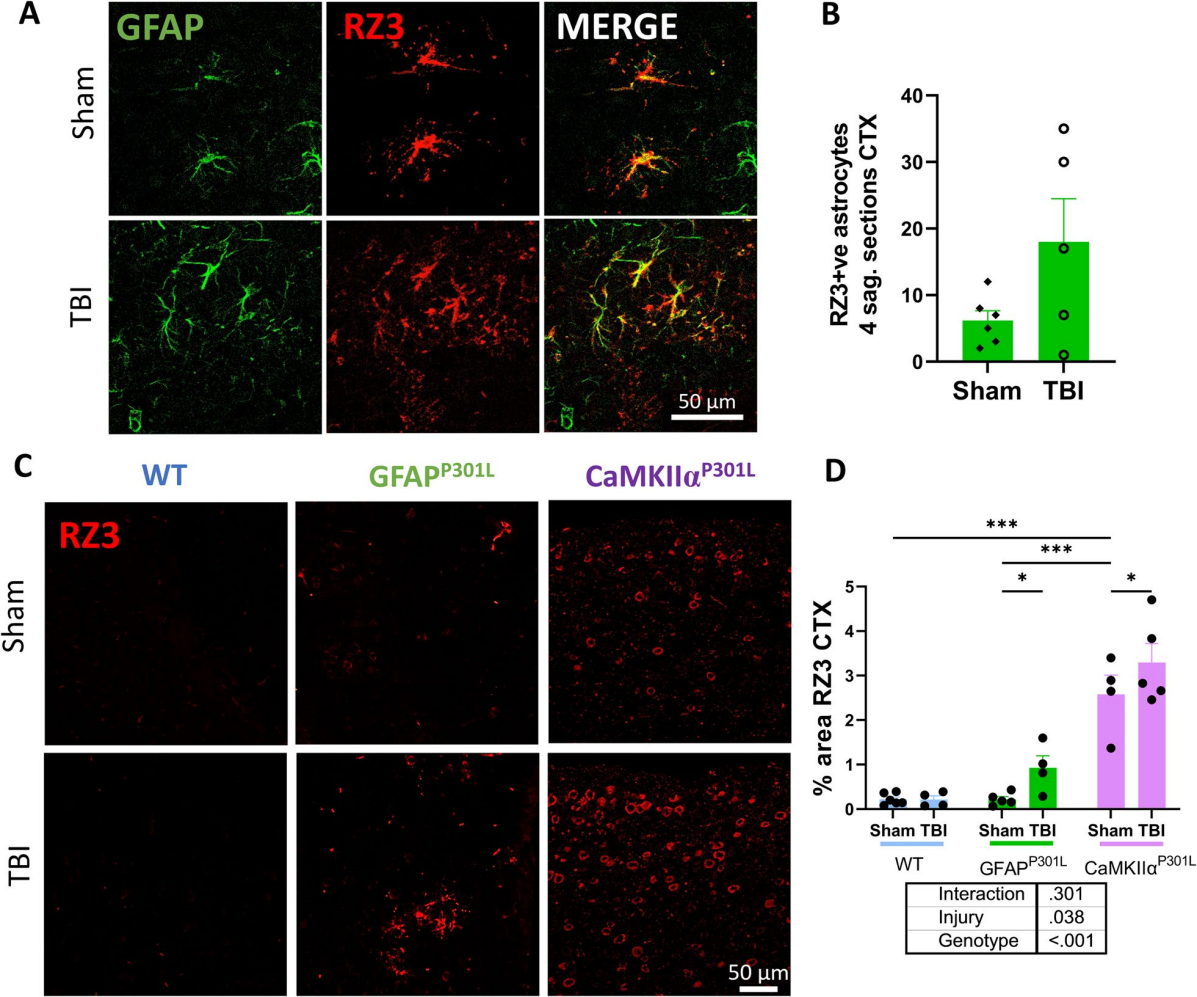
RZ3 immunoreactivity in the cortex of WT, GFAP^P301L^ and CaMKIIα^P301L^ mice at 3 months post-last injury. Qualitative images **(A)** and quantification of RZ3/GFAP + cells in the cortex of GFAP^P301L^ mice from 4 serial sagittal sections per mouse (n = 5–6 per group) 3 months post-last injury **(B)**. Qualitative images of phosphorylated tau (RZ3, red) in the cortex underneath the impact site of WT, GFAP^P301L^, and CaMKIIα^P301L^ **(C)**. RZ3 immunoreactive percent area in the cortex underneath the impact site 3 months post-last injury (n = 4–6 per group per genotype) **(D).** Data were analyzed by Two-Way ANOVA followed by the Benjamini, Krieger, and Yekuteli test. Table under the graph details injury and genotype effects and their interaction after Two-way ANOVA. Asterisks denote: *p < 0.05; **p < 0.01 and ***p < 0.001 for post-hoc analyses

To analyze brain levels of tau pathology in the regions of interest we selected antigens against several epitopes of tau hyperphosphorylation and aggregation including tau phosphorylated at Threonine 231 (RZ3), Serine 202 (CP13) and Serine 396/Serine 404 (PHF1) [23, 24]. First, we examined the effects of astroglial vs neuronal mutant human tau (htau^P301L^) on promoting tau phosphorylation in cortical astrocytes under normal circumstances. We quantified the number of astrocytes double labeled for GFAP^+^/RZ3^+^ in 4 sagittal sections of the cortex per mouse from each of the three mouse models examined in this study. GFAP^+^/RZ3^+^ astrocytes were not seen in WT and CaMKIIα^P301L^ mice. In the GFAP^P301L^ mice, an average of 6 ± 1.4 GFAP^+^/RZ3^+^ astrocytes were found in the cortex. Three months post-last TBI, the number of GFAP^+^/RZ3^+^ astrocytes remained unchanged compared to their shams (Fig. 3A, B). Next, we assessed Threonine 231 (RZ3) immunoreactivity in the cortex beneath the impact size of each mouse model. Under normal circumstances, the immunoreactivity of this tau epitope was unchanged in the GFAP^P301L^ mice compared to the WT mice (Fig. 3C, D). However, there was a significant ~ 12-fold increase in CaMKIIα^P301L^ mice compared to both WT and GFAP^P301L^ (Fig. 3D). Three months post-last injury (3mpi), there were no changes in RZ3 immunoreactivity in the WT cohort; however, the presence of astroglial pathogenic tau (GFAP^P301L^) and neuronal pathogenic tau (CaMKIIα^P301L^) mice resulted in a significant increase in p-tau (RZ3) immunoreactivity around the impact site compared to their respective sham counterparts (Fig. 3D) (two-way ANOVA: genotype effect [F(2,22) = 62.53, p ≤ 0.001]; injury effect [F(1,22) = 4.87, p = 0.038], injury*age interaction [F(2,22) = 1.27, p = 0.301]). Because tau astrogliopathy in the entire cortex did not change, the observed changes in tau phosphorylation underneath the impact site may occur in neurons.

Next, we used immunoblotting to evaluate brain levels of tauopathy by assessing tau phosphorylation markers (RZ3, CP13 and PHF1) and total tau (DA9) in the hippocampus. Because tau abundance in the CaMKIIα^P301L^ model was disproportionately increased compared to the other models, we performed a separate two-way analysis using WT and GFAP^P301L^ data only, for all markers of interest.

**Fig. 4:**
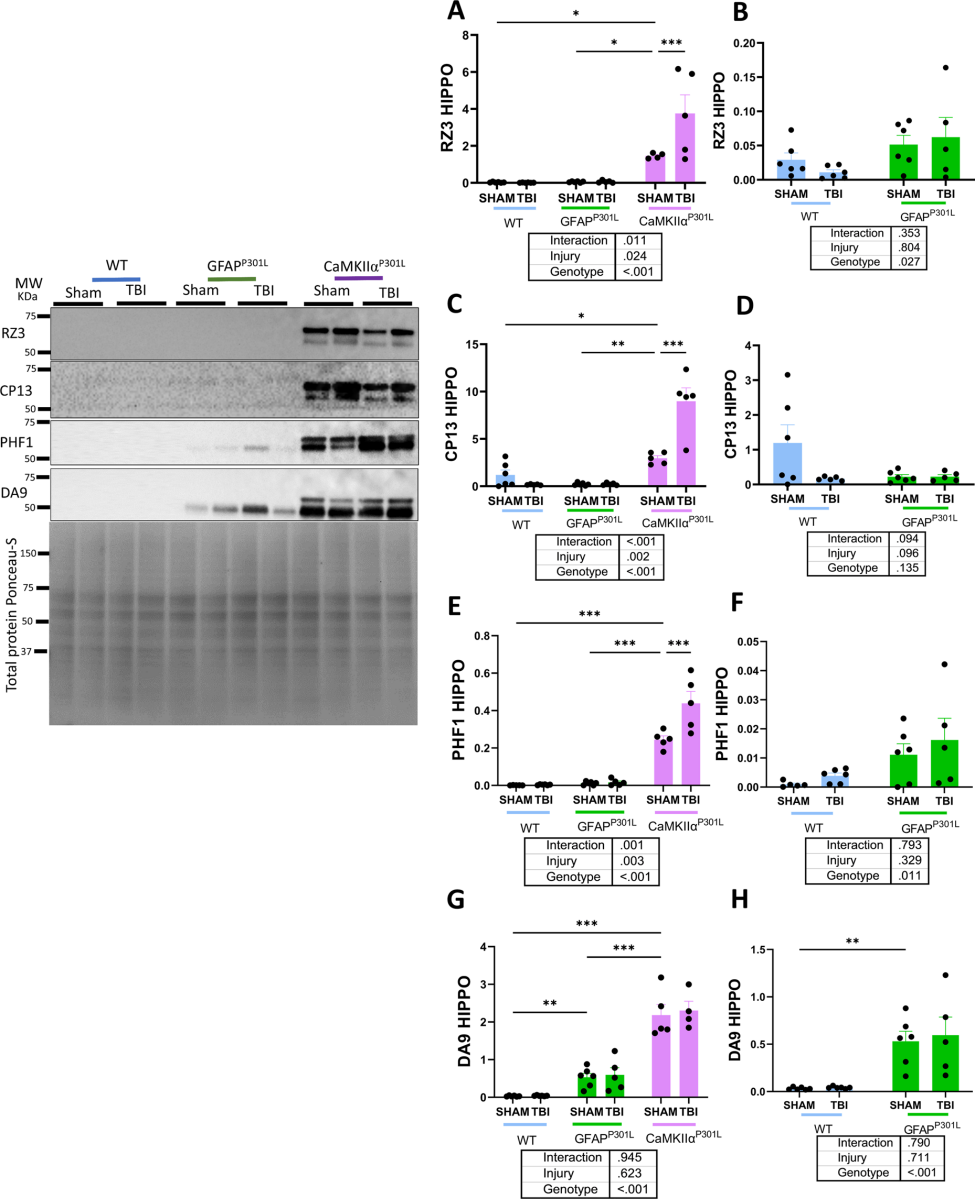
Changes in Tau species (phosphorylated and total tau) in the hippocampus of WT, GFAP^P301L^ and CaMKIIα^P301L^ mice at 3 months post-last injury. Levels of RZ3 **(A, B)**, CP13 **(C, D)**, PHF1 **(E, F)** and DA9 **(G, H)** in the hippocampus (HIPPO) at 3 months post-last injury (n = 5–6 per group per genotype). Data were analyzed by Two-Way ANOVA followed by the Benjamini, Krieger, and Yekuteli test. Table under the graph details injury and genotype effects and their interaction after Two-way ANOVA. Asterisks denote: *p < 0.05; **p < 0.01 and ***p < 0.001 for post-hoc analyses. Graphs from **B, D, F **and** H** are from WT and GFAP-P301L cohorts alone. Representative immunoblots from the hippocampus are depicted on the left of the graphs

In the hippocampus, under normal circumstances, total tau levels displayed a significant 68-fold increase in CaMKIIα^P301L^ compared to WT (two-way ANOVA: genotype effect [F(2,26) = 101.2, p ≤ 0.001]) (Fig. 4G) and a significant 18-fold increase in GFAP^P301L^ compared to WT mice (two-way ANOVA: genotype effect [F(1,19) = 28.19, p ≤ 0.001]) (Fig. 4H). CaMKIIα^P301L^ mice have around 4 times more levels of tau compared to GFAP^P301L^.

Three months post-last injury, in the hippocampus, r-mTBI does not cause changes in tau phosphorylation levels at any epitope in WT and GFAP^P301L^ mice. In contrast, in CaMKIIα^P301L^ mice, r-mTBI causes an increase of tau phosphorylation levels at specific epitopes, but not total tau compared to their sham controls (Fig. 4A–H) (two-way ANOVA: RZ3 injury effect [F(1,26) = 5.76, p = 0.024]; genotype*injury [F(2,26) = 5.36, p = 0.011]; CP13 [F(1,26) = 11.65, p = 0.002]; genotype*injury [F(2,26) = 19.74, p = 0 < 0.001]; PHF1 [F(1,26) = 10.44 p = 0.003]; genotype*injury [F(2,26) = 8.97, p ≤ 0.001]; DA9 [F(1,26) = 0.24, p = 0.623]). The lack of a TBI-mediated effect on tau phosphorylation in the hippocampus may indicate dilution of the signal on regions distal to the site of impact.

**Fig. 5:**
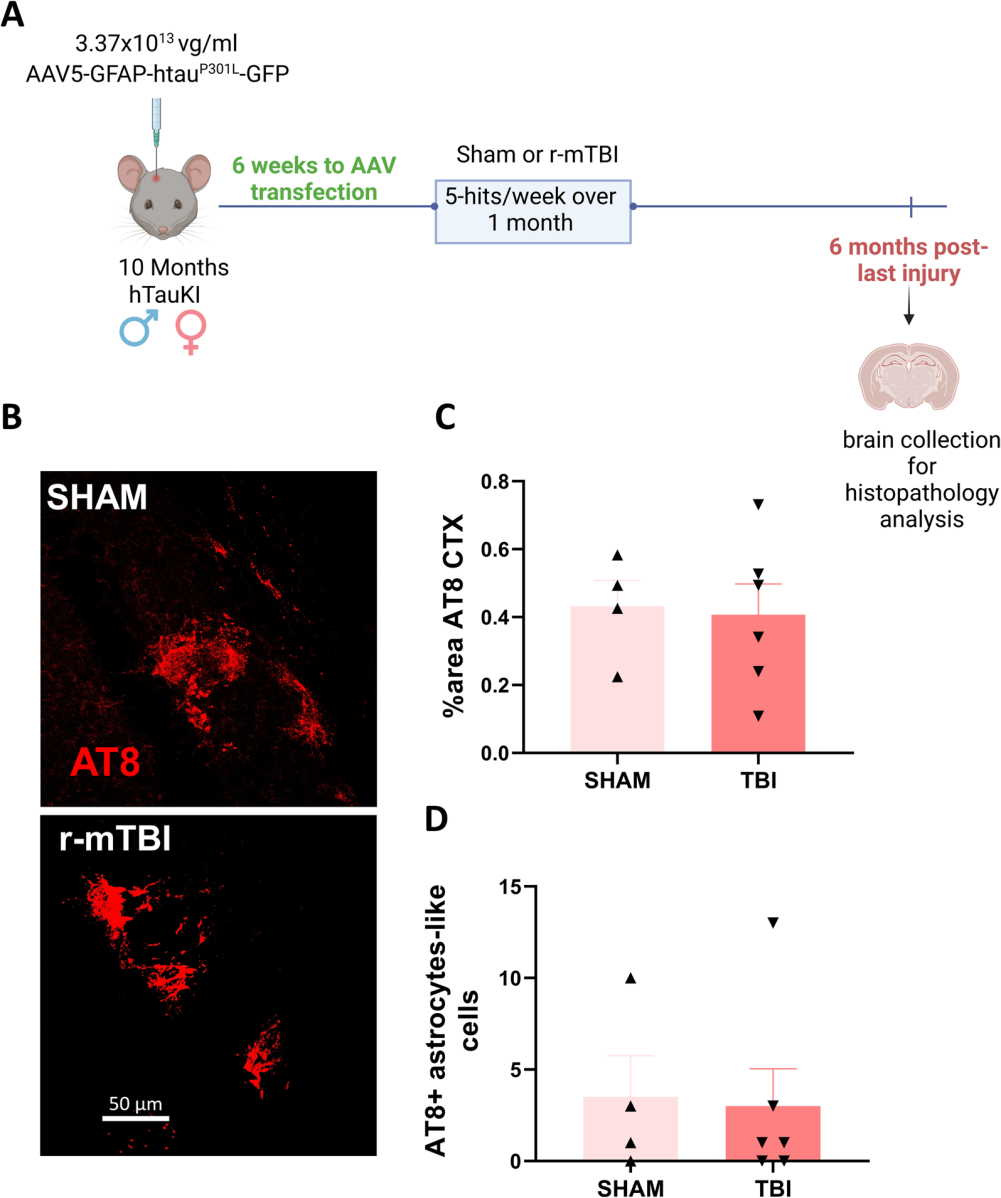
Adeno-associated viral (AAV) mediated transfection of astrocytes with mutant P301L tau prior to r-mTBI/sham injuries in human Tau Knock In (TauKI) mice. Schematic representation of AAV study design involving transfection of astrocytes under GFAP promoter with an intracerebral injection of AAV-GFAP-eGFP-P301L-Tau-FlagTau vector, six weeks prior to exposure to r-mTBI/sham injuries in 10-month-old TauKI mice **(A)**. Qualitative micrographs of phosphorylated tau (AT8) marker by immunofluorescence 6 months post-last injury **(B).** AT8 immunoreactivity in the cortex of injected naïve mice (n = 4–6 per group) **(C).** AT8 + astrocyte-like cells in the cortex of injected mice (n = 4–6 per group) **(D).** t-test analysis yielded no significant changes

Lastly, we wanted to overcome any developmental issues related to astrocytic tau production from birth in the GFAP^P301L^ model, by utilizing astrocyte-targeted AAV transfection of the mutant tau. Six weeks after transfection we confirmed mutant tau expression within astrocytes, and mice were subjected to our 20hit r-mTBI model (Fig. 5A). Then we evaluated tau phosphorylation levels in the ipsilateral cortex across 7 coronal sections at a more chronic time post last injury (6mpi) to possibly capture the progressive nature and of tau phosphorylation spread. We observed that tau phosphorylation across the cortex and the number of cortical astrocyte-like cells that were positive for phosphorylated tau (AT8^+^) remained unchanged in sham and TBI mice after transfection (Fig. 5B–D). Thus, we corroborated in mice, independent of the time of astrocytic tau overexpression, TBI does not cause cortical-wide neuronal tauopathy or exacerbate tau astrogliopathy in the cortex, even by 6 months post-injury.

### Effects of astrocytic versus neuronal tau on astrocyte and microglial reactivity in cortex under normal circumstances and following r-mTBI

**Fig. 6:**
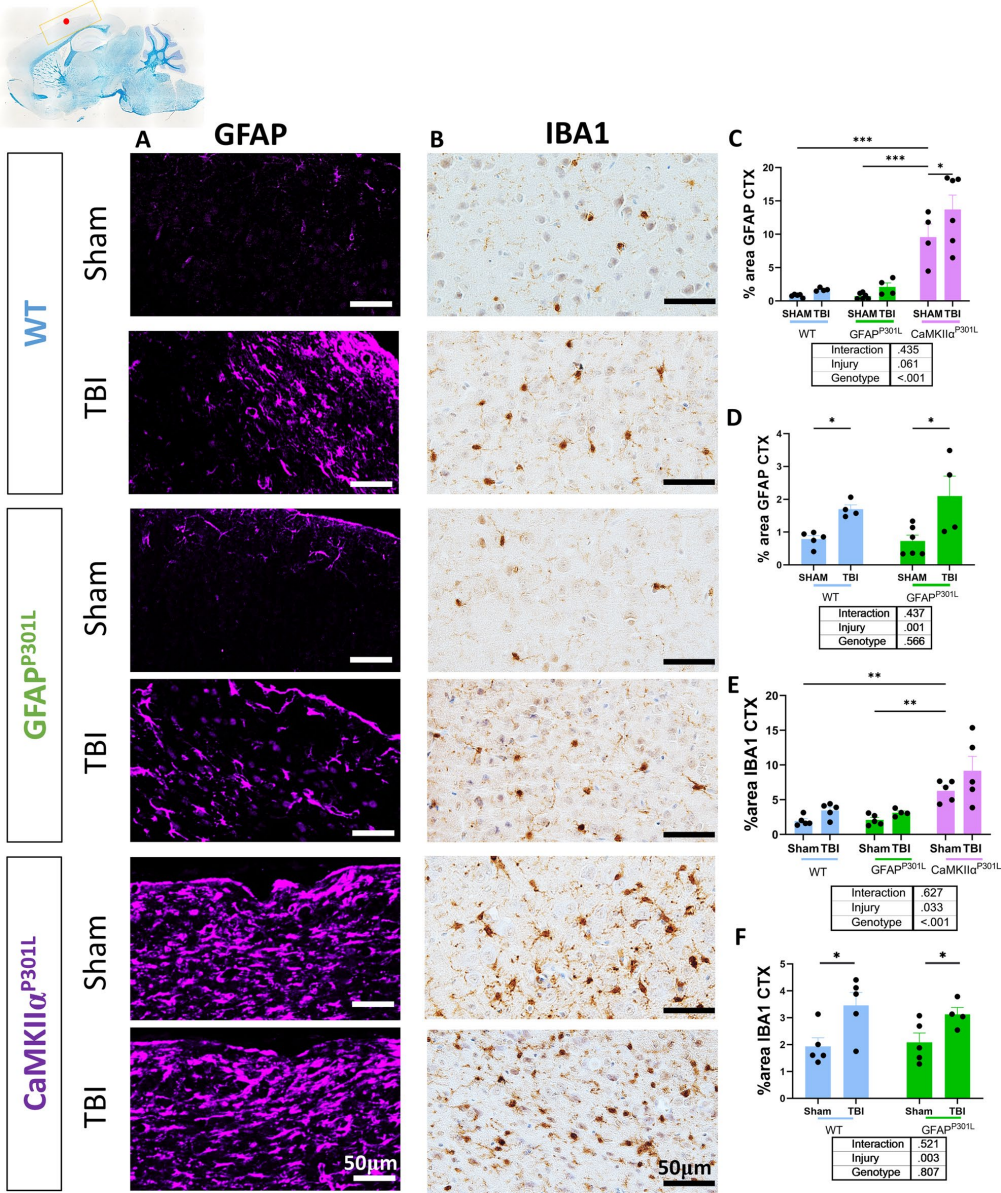
Astrocyte reactivity (GFAP) and microglial reactivity (Iba1) in the cortex (CTX) of WT, GFAP^P301L^ and CaMKIIα^P301L^ mice 3-months after r-mTBI/sham injury. Top right image is the overview of the region of interest (yellow box) where the images were collected from (red dot indicates the impact site). Qualitative images of GFAP and Iba1 in the cortex (**A** and **B**, respectively) of WT mice (top-two panels), GFAP^P301L^ mice (middle-two panels) and CaMKIIα^P301L^ mice (bottom-two panels) 3-months after r-mTBI/sham injury. GFAP images were captured at × 20 magnification and IBA1 images at x40 magnification. Percentage area of GFAP (**C, D**) and Iba1 (**E, F**) in the cortex tissue (n = 4–6 per group per genotype). Data were analyzed by Two-Way ANOVA followed by the Benjamini, Krieger, and Yekuteli test. Table under the graph details injury and genotype effects and their interaction after Two-way ANOVA. Asterisks denote: *p < 0.05; **p < 0.01 and ***p < 0.001 for post-hoc analyses. Graphs from **D** and** F** are from WT and GFAP-P301L cohorts alone

To investigate the effect of astrocyte-derived and neuronal-derived tau on astrocyte and microglial morphological phenotype at baseline and following r-mTBI, we quantified the percent area of GFAP and Iba1 immunoreactivity to evaluate astrocyte and microglial reactivity, respectively, in the cortex of each mouse model (Fig. 6A–F). Under normal circumstances, the presence of tau within astrocytes in the GFAP^P301L^ model did not impact GFAP and Iba1 immunoreactivity levels in the cortex (Fig. 6C–F) compared to WT. The presence of neuronal mutant tau in the CaMKIIα^P301L^ mice led to a significant tenfold increase in GFAP immunoreactivity in the cortex (two-way ANOVA: genotype effect [F(2,23) = 40.54, p ≤ 0.001]), compared to both WT and GFAP^P301L^ mice (Fig. 6E, I). Iba1 immunoreactivity in the cortex was significantly upregulated compared to the other two genotypes in the CaMKIIα^P301L^ mice, represented by a twofold increase (two-way ANOVA: genotype effect [F(2,23) = 28.55, p ≤ 0.001]) (Fig. 6E, F).

Three months post-last injury, there was a significant 1.5-fold increase in GFAP immunoreactivity in the cortex of the CaMKIIα^P301L^ r-mTBI group compared to sham controls (two-way ANOVA: injury effect [F(1,23) = 3.88, p = 0.061]; genotype*injury [F(2,23) = 0.86, p = 0.435]) (Fig. 6E). Because astroyte reactivity in the CaMKIIα^P301L^ model (both r-sham and r-mTBI mice cohorts) was disproportionately exacerbated compared to the other models, we performed a separate two-way analysis using only the r-mTBI/sham WT and GFAP^P301L^ data (see Fig. 6D). Our analysis revealed that r-mTBI induced a significant twofold increase in GFAP immunoreactivity in both WT and GFAP^P301L^ cohorts compared to sham counterparts (two-way ANOVA: injury effect [F(1,15) = 15.62, p = 0.001]; genotype*injury [F(1,15) = 0.63, p = 0.437]). Unlike exposure to exogenous tau (i.e., CaMKIIα^P301L^ model), the presence of tau within astrocytes (i.e., the GFAP^P301L^ model) was not sufficient to evoke an exacerbated TBI response compared to WT counterparts. Furthermore, no synergistic TBI effect was found in the GFAP^P301L^ cohort compared to WT counterparts.

Iba1 immunoreactivity demonstrated no significant changes in the CaMKIIα^P301L^ cohort in the TBI group compared to shams (two-way ANOVA: injury effect [F(1,23) = 5.14, p = 0.033]; genotype*injury [F(2,23) = 0.47, p = 6.27]) (Fig. 6E). Microglial reactivity was significantly increased in the TBI groups of both models compared to sham counterparts (two-way ANOVA: injury effect [F(1,15) = 11.98, p = 0.003]; genotype*injury [F(2,15) = 0.432, p = 0.521]) (Fig. 6F). The effect of r-mTBI exposure was not worsened in the GFAP^P301L^ cohort compared to WT.

These data indicate presence of pathological tau within astrocytes does not exacerbate the effect of r-mTBI on chronic astrocyte and microglial reactivity at 3 months post-injury. However, r-mTBI elicited an augmented glial response in CaMKIIα^P301L^ mice at 3 months post-last injury.

### Effects of astrocytic versus neuronal tau on astrocyte homeostatic markers in the hippocampus under normal circumstances and following r-mTBI

**Fig. 7:**
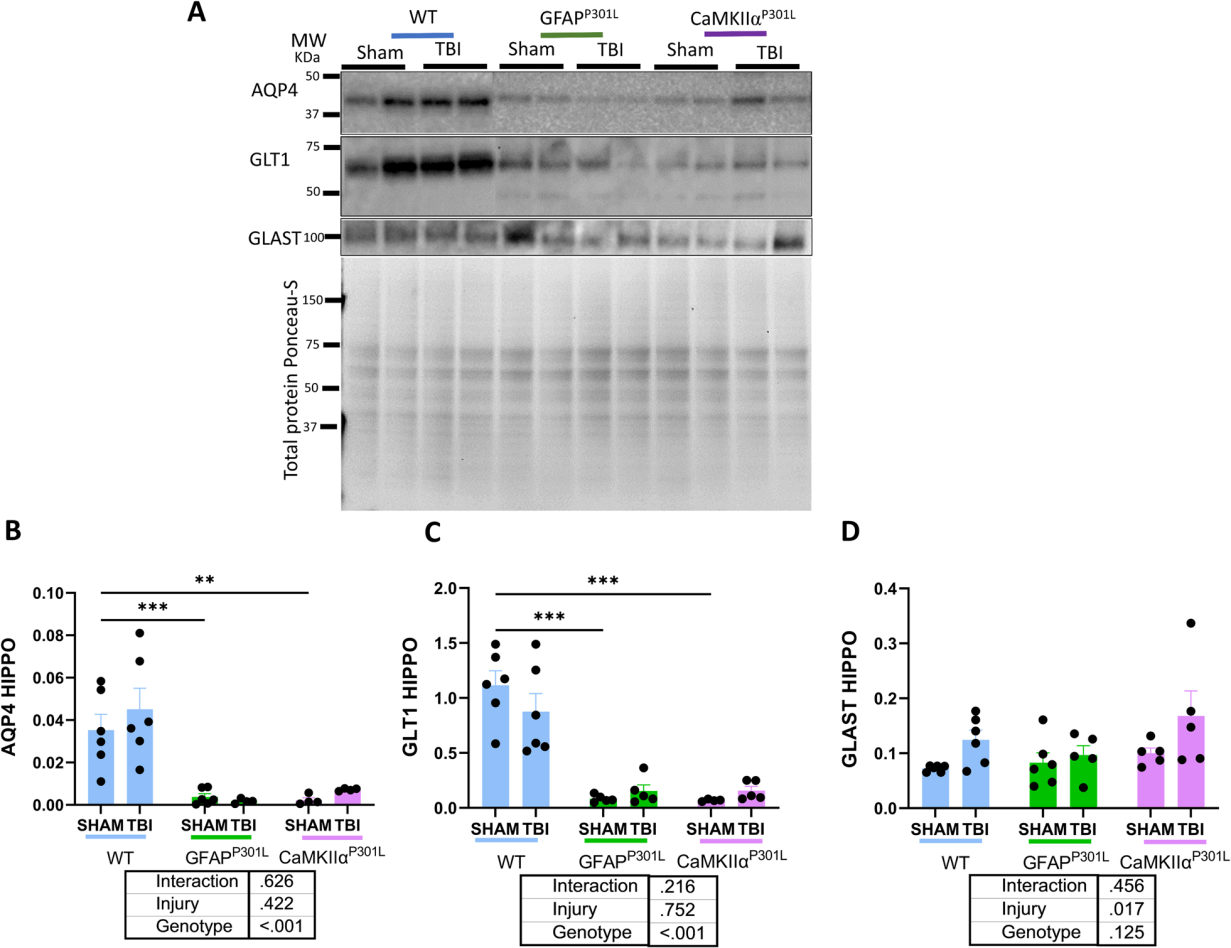
Changes in astrocyte homeostatic protein markers in WT, GFAP^P301L^ and CaMKIIα^P301L^ mice at 3 months post-last injury. Qualitative (**A**) and quantitative immunoblotting levels of aquaporin 4 (AQP4) (**B**) and glutamate transporters GLT1 and GLAST (**C, D**) in the hippocampus (HIPPO) (n = 4–6 per group per genotype). Data were analyzed by Two-Way ANOVA followed by the Benjamini, Krieger, and Yekuteli test. Table under the graph details injury and genotype effects and their interaction after Two-way ANOVA. Asterisks denote: *p < 0.05; **p < 0.01 and ***p < 0.001 for post-hoc analyses

Under normal circumstances, a significant reduction in AQP4 and GLT1 protein levels was observed in the hippocampus of both tau models (GFAP^P301L^ and CaMKIIα^P301L^) compared to WT (Fig. 7A–C) (two-way ANOVA: genotype effect [F(2,24) = 24.43, p ≤ 0.001]; genotype effect [F(2,25) = 49.14, p ≤ 0.001], respectively). GLAST levels in the hippocampus remained unchanged across genotypes (Fig. 7D) (two-way ANOVA: genotype effect [F(2,27) = 2.25, p = 0.125]).

Three months post-last injury, analyses revealed an absence of TBI-dependent changes in the levels of AQP4, GLT1, and GLAST in both regions of interest across the three genotypes (Fig. 7) (two-way ANOVA: injury effect [F(1,24) = 0.668, p = 0.422]; injury effect [F(1,25) = 0.10, p = 0.752]; injury effect [F(1,27) = 6.42, p = 0.017], respectively, none of them had genotype injury interaction).

### Astrocyte-specific transcriptional changes in response to astroglial vs neuronal tau under normal circumstances

**Fig. 8:**
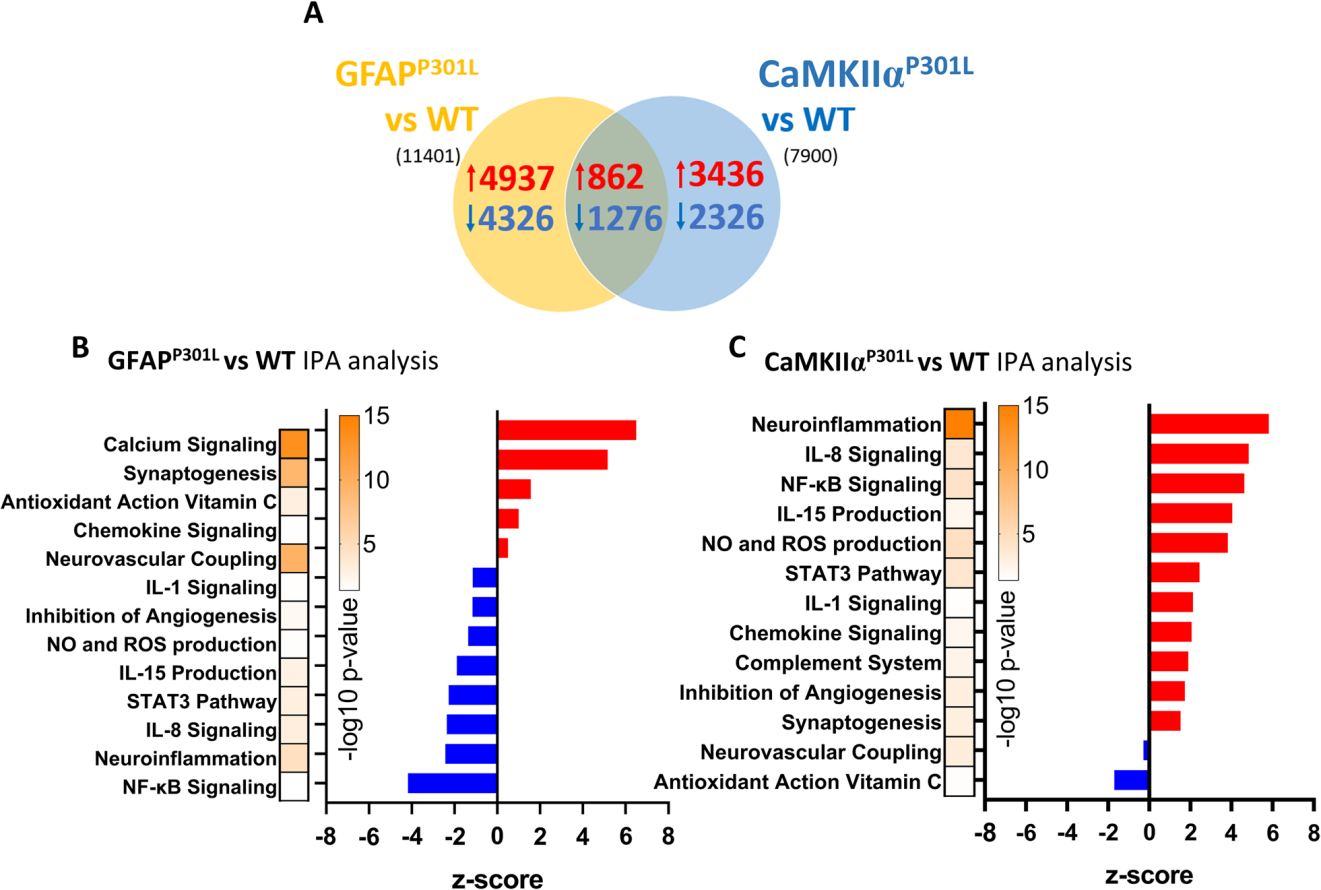
Astrocyte specific pathways that are dysregulated in GFAP^P301L^ and CaMKIIα^P301L^ mice compared to Wild-type mice at 7-month-old. Venn diagram of differentially expressed genes (DEGs) of primary astrocytes isolated using MACS ACSA2 + beads from our mouse models are shown in **A**. Histogram in **B** and **C** depicts results of IPA pathway analyses after analyzing DEGs between GFAP^P301L^ vs WT and CaMKIIα^P301L^ vs WT, respectively. Upregulated and downregulated pathways in **B–C** are depicted in red and blue, respectively. Heat-bar in **B–C** represents –log 10 of the p value (yellow—Topmost significant; purple—least significant). Threshold for obtaining the DEGs: adj. p-value ≥ 0.05 with its respective –log value ≥ 1.3. N = 3 per group per genotype

Through cell-specific bulk RNA sequencing of astrocytes harvested from our three mouse models we were able to evaluate unbiased changes in gene expression and dysregulation of cellular pathways. First, we verified that our samples contained astrocytes by assessing the transcriptional expression of several published astrocyte-specific markers [25]. A clear enrichment of protein and transcriptomic astrocytic markers compared to those for microglia, endothelial cells, oligodendrocytes and neurons was found. Interrogating significantly altered genes (adjusted p-value < 0.05) in the GFAP^P301L^ model, the presence of tau within astrocytes dysregulated the expression of 11,401 genes (5799 upregulated DEGs and 5602 downregulated DEGs) compared to WT mice. In comparison, the presence of neuronal tau in CaMKIIα ^P301L^ astrocytes dysregulated 7900 genes (4298 upregulated DEGs and 3602 downregulated DEGs) compared to WT astrocytes (Fig. 8A).

Pathway enrichment analysis was performed on DEGs (adjusted p-value < 0.05 and IPA fold change cut-off =  ± 1.2). Under normal conditions, interestingly, two opposing astroglial profiles were observed. Tau-bearing astrocytes show an immunosuppressed phenotype, characterized by the downregulation of *interleukin signaling (IL-8, IL-15, IL-1), NFκB and STAT3 signaling*, as well as *production of nitric oxide (NO), and reactive oxygen species (ROS)* (compared to WT astrocytes (Fig. 8B). On the contrary, CaMKIIα^P301L^ astrocytes displayed upregulation of the above-mentioned pathways compared to WT astrocytes (Fig. 8C). Key pathways identified in GFAP^P301L^ astrocytes compared to WT include upregulation of *calcium signaling,* and *antioxidant action of Vitamin C (ascorbic acid)* compared to WT astrocytes. CaMKIIα^P301L^ astrocytes also exhibited a downregulation of *neurovascular coupling* and *antioxidant action* transcripts. Together these findings suggest that GFAP^P301L^ astrocytes have an immunosuppressed phenotype with an enhanced antioxidant role and dysregulated calcium signaling, while CaMKIIα^P301L^ astrocytes have a proinflammatory phenotype and an impairment in antioxidant defense.

To identify DEGs common to astrocytes expressing human pathogenic tau (GFAP^P301L^) and those exposed to neuronal-derived htau^P301L^ (CaMKIIα^P301L^), we queried DEGs obtained from GFAP^P301L^ versus WT and CaMKIIα^P301L^ versus WT mice. This comparison found 2,138 convergently dysregulated genes (862 upregulated DEGs and 1,276 downregulated DEGs) (Fig. 8A). Enriched pathway analysis identified that those convergent DEGs were associated with the upregulation of *chemokine signaling, synaptogenesis, neuroinflammation, and mitochondrial dysfunction*; and the downregulation of *neurovascular coupling* and *detoxification function (glutathione-mediated).*

### Astroglial transcriptome changes in response to r-mTBI three months post-last hit

**Fig. 9:**
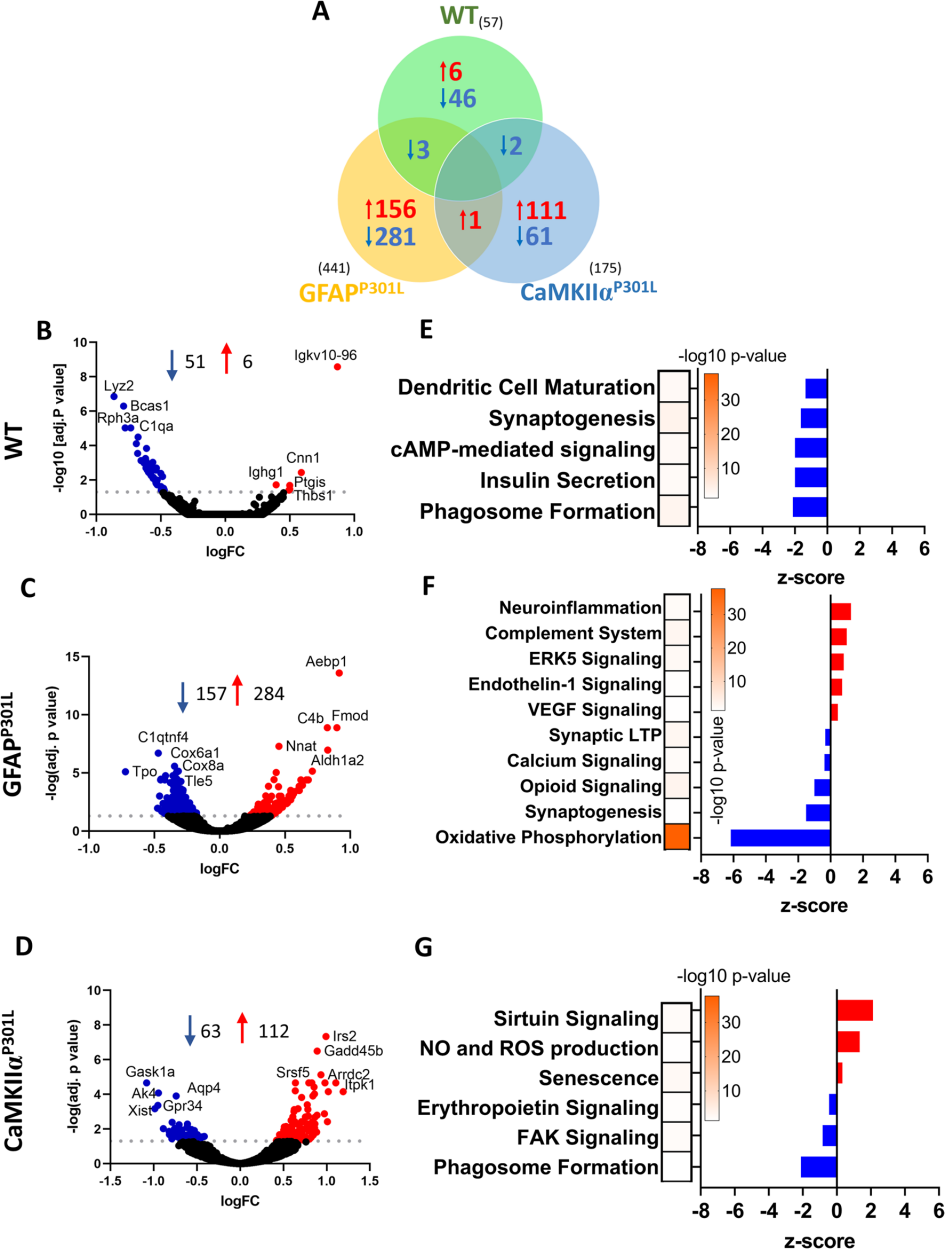
Astrocyte specific pathways that are dysregulated in WT, GFAP^P301L^ and CaMKIIα^P301L^ mice at 3-month post-last injury. Venn diagram of injury dependent differentially expressed genes (DEGs) primary astrocytes isolated using MACS ACSA2 + beads from our mouse models are shown in **A** (i.e., entire DEGs, overlapping DEGs and unique DEGs). Volcano plot of injury dependent DEGs are shown in **B** (WT), **C** (GFAP^P301L^) and **D** (CaMKIIα^P301L^). Top 10 DEGs are highlighted on the volcano plots. Upregulated DEGs are in red, Downregulated DEGs are in blue. Histogram in **E** (WT)**, F** (GFAP^P301L^) and **G** (CaMKIIα^P301L^) depicts results of IPA pathway analyses after analyzing the entire DEG list between r-mTBI vs sham groups for each of the 3 different genotypes. Upregulated and downregulated pathways in **E–G** are depicted in red and blue, respectively. Heat-bar in **E–G** represents -log 10 of the p value (yellow—Topmost significant; purple—least significant). Threshold for obtaining the DEGs: adj. p-value ≥ 0.05 with its respective –log value ≥ 1.3. N = 3 per group per genotype

Three months post-last injury (3mpi), astrocyte-specific bulk RNA analysis showed that r-mTBI caused dysregulation of 57 genes (51 downregulated DEGs and 6 upregulated DEGs) in WT, 175 (63 downregulated DEGs and 112 upregulated DEGs) in CaMKIIα^P301L^, and 441 genes (157 downregulated DEGs and 284 upregulated DEGs) in GFAP^P301L^ mice (Fig. 9A–D). The presence of endogenous htau^P301L^ in astrocytes results in greater transcriptomic dysregulation in response to r-mTBI compared to the exposure of astrocytes to exogenous neuronal tau in the CaMKIIα^P301L^ or the absence of pathogenic human tau in the WT mice. No single gene was dysregulated across cohorts in response to TBI (Fig. 9A) highlighting the context-dependent nature of astroglial response.

IPA analysis interrogating the 57 DEGs in the WT mice revealed significant downregulation in 5 pathways: *phagosome formation, insulin secretion, c-AMP signaling, synaptogenesis and dendritic cell maturation* (Fig. 9E). In the GFAP^P301L^ cohort, 441 DEGs mapped to 52 significant pathways included the downregulation of *oxidative phosphorylation*, *synaptic long-term potentiation*, and *opioid signaling*; and upregulation of *neuroinflammation*, *complement system signaling*, and *ERK5-mediated cell activation* (Fig. 9F). In the CaMKIIα^P301L^ cohort, 175 DEGs mapped to 17 significant pathways including downregulation of *phagosome formation and neuroprotective erythropoietin signaling,* and upregulation of *nitric oxide and reactive oxygen species production*, and *senescence* (Fig. 9G). These observations revealed that r-mTBI affects GFAP^P301L^ astrocytes by compromising mitochondrial function and neuronal support mechanisms, which favors a pro-inflammatory state compared to WT and CaMKIIα^P301L^ mice.

### Transcriptome changes in astroglia obtained from human CTE tissue

**Fig. 10:**
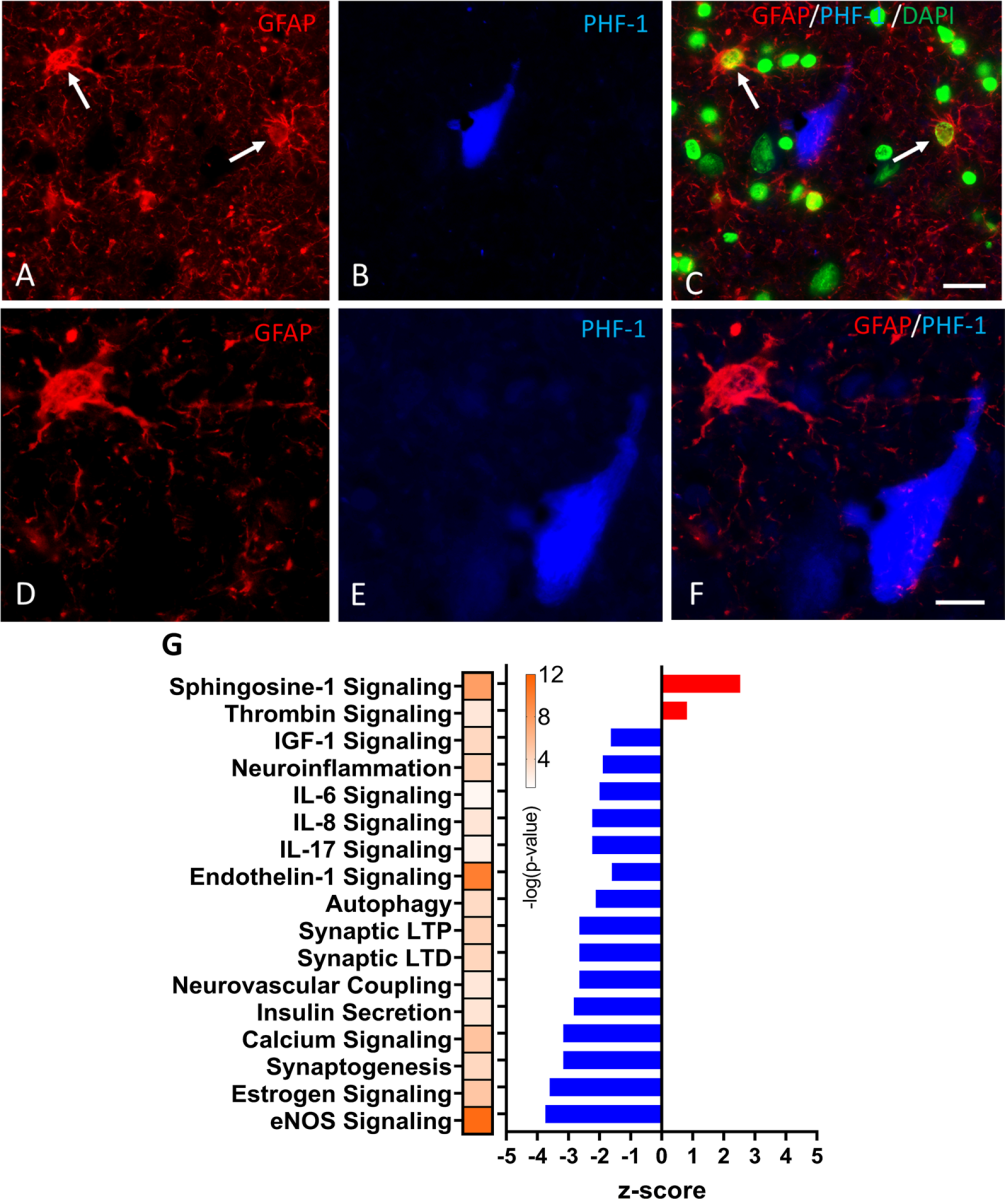
Absence of tau astrogliopathy in CTE-IV hippocampal astrocytes and astrocyte-specific pathways that are dysregulated in human CTE (stage IV) cases versus healthy controls after laser microdissection of GFAP + astrocytes and gene array analyses. Immunofluorescent label demonstrating a lack of colocalization between tau-bearing neurofibrillary tangles and GFAP^ve^ astrocytes in the CA1 region of the hippocampus of a male Caucasian American football player that played for 25 years had an age of onset of symptoms at 66 years and died in his 70s. Postmortem neuropathologic diagnosis revealed CTE stage IV. Low power Immunofluorescence images showing single labeled GFAP astrocytes (red) and PHF-1 (tau phosphorylated at S396/404) positive NFTs (blue) and a merged image combined with staining for cell nuclei (green) in the hippocampus of the CTE stage IV case **(A, B)**. High-power images of the GFAP astrocyte (upper left panel **A**, white arrow) and the NFT **(D–F)**. Scale bar = 25 μm in **A-C**; scale bar = 10 μm in **D**–**F**. Histogram depicts the results of IPA pathway analyses after analyzing the entire DEG list between CTE stage IV (n = 11) vs HC (n = 9) **(G).** Upregulated and downregulated pathways are depicted in red and blue, respectively. Heat-bar represents -log 10 of the P value (yellow—Topmost significant; dark blue—least significant). The threshold for obtaining the DEGs: adj. p-value ≥ 0.05 with its respective –log value ≥ 1.3. Approximately 50–100 astrocytes were micro-dissected and subjected to customized gene array analyses to interrogate > 850 genes with > 20 gene ontology groups

Prior to performing gene array analysis, we used dual immunofluorescence to determine whether GFAP-stained hippocampal astrocytes colocalized with PHF-1 tau in individuals with a postmortem neuropathological diagnosis of stage IV CTE (CTE-IV). Qualitative analysis showed that hippocampal CTE-IV astrocytes did not contain PHF-1 phosphorylated tau (Fig. 10A–F). Then, we evaluated changes in gene expression in hippocampal astrocytes from CTE-IV brains compared to hippocampal astrocytes from non-demented healthy controls (HC). GFAP^+ve^ astrocytes microaspirated from CTE-IV brains revealed 156 DEGs (153 downregulated and 3 upregulated) (Fig. 10G). Pathway enrichment analysis by IPA on DEGs revealed CTE-IV astrocytes had 100 dysregulated pathways including upregulation of *thrombin signaling,* and *sphingosine signaling*, and downregulation of *neuroinflammation, interleukin signaling (IL-6, 8, 17), endothelin 1 signaling, calcium signaling, synaptogenesis, and insulin-like growth factor signaling.* These data indicate that CTE-IV astrocytes exhibit a loss of neurorestorative function(s) accompanied by an immunosuppressed phenotype compared to healthy controls. Interestingly, despite that CTE-IV hippocampal astrocytes do not display phosphorylated tau (PHF1) in their soma, CTE-IV astrocytes showed significant downregulation of genes associated with immunological response including *IL-8 signaling and NO and ROS production* similar to tau bearing murine astrocytes (GFAP^P301L^).

## Discussion

Tau astrogliopathy is a pathological feature of primary tauopathies including CTE. However, the role that tau astrogliopathy plays in the onset and/or progression of TBI/CTE pathophysiology remains underinvestigated. Using the GFAP^P301L^ mouse model harboring tau-bearing astrocytes, we investigated the effects of endogenous htau^P301L^ accumulation, r-mTBI and how their interaction might affect astroglial pathological response. Our findings reveal that astroglial overexpression of htau^P301L^ does not alter markers of gliosis but elicits a significant reduction in hippocampal homeostatic astrocyte-specific markers (AQP4 and GLT1) that regulate water transport and glutamate homeostasis, respectively. Additionally, astrocytes overexpressing tau display an immunosuppressed phenotype similar to the phenotype observed in stage IV CTE-derived astrocytes. Moreover, r-mTBI does not augment or accelerate tau astrogliopathy or gliosis but causes an increase in neuronal phosphorylated tau in the cortical area underneath the impact site and astroglial mitochondrial dysfunction.

Pathological analyses of human CTE cases show significant increases in gliosis at the interface between white and grey matter [26]. The present findings indicate that compared to astrocytic tau overexpression, neuronal tau elicits significantly more astrogliosis and microgliosis in the cortex grey matter compared to WT mice and in the white matter of the corpus callosum (supplementary Fig. 1). At the age of 7 months (mice age at 3mpi), CaMKIIα^P301L^ mice already exhibit substantial tau pathology [27] while increased tau pathology in GFAP^P301L^ mice does not occur until 24 months [5]. Foreman and colleagues reported that in regions of the brain where tau astrogliopathy was robust, astrogliosis was also exacerbated. Together with the present findings, the appearance of tau astrogliopathy at 7mo of age is not sufficient to increase gliosis. Since our preliminary data (supplementary Fig. 2) and findings from other models of tauopathy [4, 5] suggest that GFAP^P301L^ mice do not exhibit robust astroglial tauopathy earlier than 24 months of age. Therefore, we combined r-mTBI (20-hit paradigm) with the astroglial tau model to evaluate the onset and/or progression of tau astrogliopathy. Repetitive mTBI evoked a region-specific (i.e., underneath the impact site) increase in cortical levels of tau phosphorylation at 3mpi but it did not affect cortical-wide tau phosphorylation at 3 and 6mpi. Furthermore, the number of astrocytes containing phosphorylated tau (i.e., tau astrogliopathy) remained unchanged between sham and TBI at chronic timepoints, 3 and 6mpi. In the hippocampi of CTE-IV brains, we did not observe PHF-1 reactive astrocytes stained with GFAP, which suggests that tau astrogliopathy in the hippocampus is inconspicuous compared to neuronal tau [28, 29]. CTE pathology in the dorsal lateral frontal cortex from this brain bank has previously been shown to be primarily neuronal [28]. Further analysis with other markers of tau phosphorylation, such as AT8 or CP13, will be needed to confirm the extent of tau astrogliopathy in the hippocampus of CTE samples used for this study. Additionally, since all astrocytes do not stain with GFAP, other homeostatic markers—e.g., S100β—may be also required. Three months post-last brain injury, we detected increased astrogliosis and microgliosis in the cortex and CC beneath the impact of GFAP ^P301L^ mice (TBI vs sham) (Supplementary Fig. 1), however, TBI-induced gliosis was not exacerbated compared to WT TBI counterparts.

These results suggest that overexpression of tau ^P301L^ in astrocytes in GFAP^P301L^ mice is not a necessary precondition to cause significant gliosis compared to WT mice. Although r-mTBI did not increase tau astrogliopathy, it initiated an increase in tau phosphorylation in the cortex beneath the impact. This increase in tau phosphorylation is not robust enough to exacerbate TBI-mediated gliosis compared to the WT cohort. In the case of the CaMKIIα^P301L^ mice, the substantial presence of phosphorylated tau might underlie the greater glial response seen in this transgenic mouse than in WT mice. We suggest that TBI-dependent gliosis in humans might be orchestrated by an increase in neuronal tauopathy rather than tau astrogliopathy. Further investigation on aged GFAP^P301L^ mice could potentially aid in understanding the contribution of robust tau astrogliopathy to glial responses.

Biochemical analysis of homeostatic astrocyte markers in our mouse models of tau astrogliopathy and neuronal tauopathy revealed a region-specific reduction of AQP4 and GLT1 in the hippocampus of shams compared to WT shams. AQP4 is a water channel located on astrocytic perivascular end-feet that is essential for water flux and waste clearance termed the glymphatic system [30]. AQP4 is also present in perineuronal astrocytic processes where it is involved in maintaining water volume and ion/neurotransmitter buffering in response to synaptic transmission [31]. Skucas and colleagues (2011) reported that AQP4-/- mice have impaired memory consolidation linked to impairments in long-term potentiation and long-term depression (LTP/LTD) [32]. Based on hippocampal AQP4 downregulation in both tauopathy models, we expect GFAP^P301L^ have impaired memory. Interestingly, CamkIIα^P301L^ mice exhibit behavioral changes at the age of 5 months [33], which likely are linked to the substantial accumulation of pathogenic tau. Moreover, r-mTBI did not elicit changes in AQP4 levels in the mouse models confirming other studies that have reported no differences in AQP4 abundance after TBI [34, 35]. Glutamate transporter 1 (GLT1) an astroglial-specific marker was also affected by the presence of tau overexpression in astrocytes or neurons only in the hippocampus. GLT1 is responsible for preventing excitotoxicity by recycling 85% of glutamate excess at the synaptic cleft after synaptic transmission [36]. Thus, a significant reduction of GLT1 expression would lead to a significant increase in extracellular glutamate concentration which ultimately can cause neuronal loss [2]. Previous studies on CaMKIIα^P301L^ mice have shown a ~ 55% loss of hippocampal neurons by the age of 6 months [37, 38], which may suggest a link between a reduction of GLT1 and neuronal loss due to excitotoxicity. However, we observed no genotype or injury-related changes in markers of synaptic integrity such as synaptophysin and PSD95 in our models (data not shown) which suggests that the significant reduction in GLT1 levels might affect neurons at a functional level. Further experiments assessing glutamate concentration and electrophysiological properties of neurons in these models are needed to confirm this relationship. Moreover, r-mTBI did not cause changes in GLT1 or GLAST at 3mpi compared to controls. A previous study showed a significant reduction of GLT1 in the cortical astrocytes after TBI [39]. The authors also noted that the GLT1 downregulation was observed in astrocytes directly below the impact site. Additional experiments are essential to assess the mechanism and the functional effects of alteration in glutamate transporter and AQP4 abundance.

Pathway analysis of DEGs shows that, under sham conditions, astrocytic tau overexpression compared to neuronal tau overexpression resulted in greater transcriptional astrocyte change. Tau-bearing astrocytes (GFAP^P301L^ astrocytes) showed an immunosuppressed phenotype. On the contrary, CaMKIIα^P301L^ astrocytes exposed to neuronal tau and robust microgliosis showed a pro-inflammatory phenotype and lost their neurovascular and antioxidant function. Under injury conditions, GFAP^P301L^ astrocytes showed greater transcriptional dysregulation compared to WT and CaMKIIα^P301L^ astrocytes 3mpi.

Downregulation of neuroinflammatory pathways including IL-12 and IL-15 in GFAP^P301L^ sham astrocytes might cause suppression of astrocyte-microglia bidirectional crosstalk since microglia respond to both interleukins by mounting immune responses [40, 41]. These findings may explain the failed genotype effect observed in microgliosis and astrogliosis between GFAP^P301L^ and WT sham groups. Interestingly, CTE-IV hippocampal astrocytes (tau-negative) also manifest an immunosuppressed phenotype characterized by the downregulation of IL-8 signaling. Since IL-8 signaling is involved in microglial activation [42], its downregulation may alter the ability of astrocyte-microglia interaction needed to mount an immune response. This similarity might indicate that hippocampal human astrocytes and murine astrocytes share a “loss of function” trait irrespective of astrocytic tau accumulation. In contrast, Chancellor and colleagues showed that CTE white matter astrocytes exhibit downregulation of normal functioning, and dysfunctional mitochondrial metabolism accompanied by increased neuroinflammation resulting in a “neurotoxic profile” [43]. The observed discrepancies in transcriptional changes might be related to the heterogenicity of astroglial response depending on the region they are found respective to the impact site (hippocampus vs white matter). Moreover, astrocytes harvested from individuals with other neurodegenerative conditions, namely, AD, PD, MS, and HD show an upregulation of inflammatory responses [44] which contrasts with what we are reporting in the present studies. However, analogous to what is seen in neurodegenerative diseases, murine astrocytes may display a senescent phenotype following TBI characterized by the dysregulation of astrocyte-secreted molecules important for cell-to-cell communication [46]. Additionally, it has also been reported that a sub-population of astrocytes in neurodegenerative conditions can display downregulation of transcripts involved in synaptogenesis and neuronal function [44, 45] similar to the phenotype of hippocampal CTE-IV astrocytes reported here. This suggests that astrocytes might have a common pathobiological response in neurodegenerative conditions characterized by the loss of their neuronal support functions and mechanisms.

Altogether, these findings suggest that tau astrogliopathy in CTE might result in a “loss of function” phenotype where hippocampal astrocytes fail to mount an immune response, communicate to microglia, support synaptogenesis and maintain neurovascular coupling which may be involved in the neurodegenerative nature of CTE. The prominent pathways identified in the present study warrant further investigation to understand and determine if the molecular changes dictating their dysregulation might represent therapeutic targets. Given that tau-bearing astrocytes from the sham GFAP^P301L^ mice (vs WT) also showed a similar immunosuppressed state we suggest that the GFAP^P301L^ model is a relevant platform in which to study tau astrogliopathy in tauopathies.

### Limitations of the study

Data presented here provides insights into the effects of overexpression of pathogenic tau in astrocytes under normal conditions and after r-mTBI, however, this study has several limitations. The expression of pathogenic tau in astrocytes in the GFAP^P301L^ model occurs from embryonic day 14 [46] and persists throughout the animals’ lifespan, leading to an age-dependent increase in tau astrogliopathy. In humans, it has been suggested that the accumulation of tau within astrocytes is more likely to progressively appear after an encounter with a triggering event (e.g., cerebrovascular accidents, gene predisposition, head trauma) that either increases the expression and accumulation of astrocytic endogenous tau, or the internalization and aggregation of secreted neuronal tau [29].

Tau expression in our model is controlled by the GFAP promoter whose expression is region-dependent and known to increase in response to TBI [47]. This leads to differential expression/accumulation of tau within astrocytes throughout the brain which might contribute to regional astroglial response under normal (sham) conditions and r-mTBI. An increase in GFAP expression in response to TBI is likely to confound the effects of TBI alone on tau pathology in this model. In the future, we will consider the utilization of an astrocyte-specific promoter that is known to not change in response to TBI such as Aldehyde dehydrogenase 1 family, member L1 (ALDH1) or Connexin 30 (Cx30) promoter [48].

It has been reported that the integration of the tetO-MAPT*P301L transgene (~ 70 copies) into chromosome 14 results in a deletion of 244kbp in the Fgf14 (Fibroblast growing factor 14). This is particularly relevant because functional knockout of FGF14 may contribute to accelerated neuronal loss and brain atrophy in tetO-MAPT*P301L-Fgf14 models [49], and thus it cannot be assumed that mutant tau, alone, drives the reported changes in neurodegeneration. Additionally, the trans activator (tTA) transgene insertion, that drives the expression of mutant tau either in astrocytes or neurons, disrupts another five genes which, collectively, have been shown to be involved in synaptic transmission, plasticity, and neurogenesis [50, 51] which may indirectly confound our findings. However, relevant to the outcomes on this paper, we observed that hippocampal levels of AQP4 did not change while GLT1 levels seem to suffer a slight decrease (28% decrease) in tetO-MAPT*P301L compared to WT (*data not shown*). Thus the tetO*MAPT*P301L transgene insertion affects hippocampal levels of GLT1 but the overproduction of mutant tau in astrocytes or neurons is what further decreases GLT1 (93% decrease) in the GFAP^P301L^ mice. Further investigation is warranted to know the impact of these transgene insertions on other markers of normal physiology of astrocytes.

Astrocyte tau pathology in CTE has been shown to be predominantly of 4R tau isoform, therefore paralleling the phenotype in our GFAP^P301L^ model. However, the CaMKIIα^P301L^ model displays 4R-neuronal tauopathy while neuronal pathology in CTE cases is mixed (3R/4R) [52]. Although the effects of 3R and 4R tau on pathology are largely unclear, they may manifest different pathogenicity individually or synergistically. More studies are therefore needed to delineate the isoform-specific effects at the cell type level.

Human GFAP^+ve^ astrocytes were microdissected from the hippocampus of CTE-IV cases. As shown in Fig. 10A–F, GFAP^+ve^ astrocytes do not contain phosphorylated tau (PHF1), thus, it is possible that the harvested human astrocytes do not contain tau. PHF1 antibodies recognize both 3R and 4R pathogenic isoforms of tau and were selected because it is widely used for the identification of both astroglial (predominantly 4R) and neuronal (both 3R/4R) tauopathies in CTE [53–55]. As mentioned before, further investigation is warranted to know if other tau epitopes might be preferentially phosphorylated (AT8, CP13, or 4R-tau) in GFAP^+ve^ astrocytes, therefore, we cannot discard or prove that the analyzed astrocytes lack accumulation of tau. Nonetheless, we acknowledge that astrocyte pathophysiology may differ in the presence or absence of astrocytic tau accumulation. Furthermore, previous reports have shown that astrocytes exhibit not only region-dependent transcriptional changes but also differential responses to stimuli [56–59]. Human RNA data was obtained from hippocampal tau-negative astrocytes (GFAP^+ve^/PHF1^−ve^) while mouse data was obtained from tau-overexpressing astrocytes harvested from the entire brain excluding the hippocampi. Therefore, direct comparisons of our human and mouse data and/or generalization of astrocytic changes based on the hippocampal astrocyte population, here reported, should be avoided. Additionally, we acknowledge that the age-matched cohort has a sex disparity (2 males and 7 females). Thus, we compared the transcriptome profile between females and males (Supplementary Fig. 3) which did not reveal obvious differences which allowed us to move forward with the CTE vs control comparisons. Lastly, the consensus RIN value for RNA samples is ≥ 7 [60], therefore we acknowledge that the reduced RIN value (RIN ≥ 5) of our samples might have resulted in under- or overrepresentation of transcripts in the library. However, Ilumina HiSeq library used for the analysis of our samples has been extensively used for the analysis of samples with a RIN = 2 and still provides high-quality RNA sequencing results [61].

## Summary

We demonstrate r-mTBI did not significantly exacerbate tau astrogliopathy, but increased tau phosphorylation in the cortical neurons beneath the impact site. Overexpression of htau^P301L^ within astrocytes did not increase astrogliosis and microgliosis compared to WT mice. Moreover, r-mTBI did not exacerbate gliosis compared to WT cohorts. Interestingly, tau-bearing astrocytes undergo region-specific changes that alter homeostatic markers related to optimal neuronal transmission and water transport independent of TBI. Tau-bearing astrocytes have significantly more DEGs compared to WT astrocytes and astrocytes exposed to robust neuronal tau pathology and microgliosis under normal circumstances. Tau-bearing murine astrocytes exhibit an immunosuppressed phenotype that might modify bidirectional molecular communication with microglia limiting the ability to mount immune responses. At three months post-last injury, tau-bearing astrocytes display a greater transcriptional dysregulation compared to WT and CaMKIIα^P301L^ astrocytes showing a compromised bioenergetic system which might interrupt their physiological roles. Finally, CTE-IV astrocytes display an immunosuppressed phenotype similar to the effects of tau overexpression seen in astrocytes from the GFAP^P301L^ mouse model.

Collectively, the findings of this study underscore the significance of unraveling mechanisms driving tau astrogliopathy and the pathobiological consequences on the normal, injured, and tauopathic brain. Our unbiased results imply a driving role in the astroglial immune state and propagating microglial-mediated neuroinflammation observed after r-mTBI. These mechanisms may suggest targeted interventions to address the pathobiology of r-mTBI that are both tau-dependent and tau-independent.

### Supplementary Information

Suuplemenatry Material 1: Figure 1: Astrocyte reactivity (GFAP) and microglial reactivity (Iba1) in the corpus callosum (CC) of WT, GFAP^P301L^ and CaMKIIα^P301L^ mice 3-months after r-mTBI/sham injury. Top right image is the overview of the region of interest (yellow box) where the images were collected from (red dot indicates the impact site). Qualitative images of GFAP and Iba1 in the CC (A and B, respectively) of WT mice (top-two panels), GFAP^P301L^ mice (middle-two panels) and CaMKIIα^P301L^ mice (bottom-two panels) 3-months after r-mTBI/sham injury. Images were captured at x20 magnification. Percentage area of GFAP (C) and Iba1 (D) in the CC (n=5-6 per group per genotype). Data were analyzed by Two-Way ANOVA followed by the Benjamini, Krieger, and Yekuteli test. Table under the graph details injury and genotype effects and their interaction after Two-way ANOVA. Asterisks denote: *P<0.05; **P<0.01 and ***P<0.001 for post-hoc analyses.

Suuplementary Material 2: Figure 2: Tau astrogliopathy in the cortex of GFAP^P301L^ mice at 7 and 10 months of age. RZ3/GFAP+ cells in the cortex from 4 serial sagittal sections at 7 and 10 months of age (n=5-6 per group). t-test analysis yielded significant changes p<0.001***

Supplementary Material 3: Figure 3: Heat map revealing expression levels of all genes in the microarray of all healthy control (HC) cases and brief clinical demographics of CTE and HC cohorts. Heat map depicts relative intensity score (i.e., expression levels) of all genes in the microarray from all healthy control (HC) cases (n=9). Upregulated and downregulated genes are depicted in red and blue, respectively.

Supplementary Material 4: Table 1. Antibodies used. Abbreviations: GFAP, Glial Fibrillary Acidic Protein; Iba1, Ionized calcium-binding adaptor molecule; AQP4, aquaporin 4; GLAST, Glutamate transporter; GLT1, Glutamate transporter 1. IF, Immunofluorescence; IHC, Immunohistochemistry; WB, Western blotting.

## Data Availability

Data used and analyzed in the current study are available upon request from the corresponding author.

## Abbreviations

AD: Alzheimer’s disease
ALDH1: Aldehyde dehydrogenase 1 family, member L1
ANOVA: Analysis of variance
AQP4: Aquaporin 4
ARTAG: Age-related tau astrogliopathy
BBB: Blood–brain barrier
CaMKIIα: Calcium/calmodulin-dependent protein kinase II alpha
CBD: Cortico-basal degeneration
CC: Corpus callosum
CNS: Central Nervous System
CTE: Chronic Traumatic Encephalopathy
CTX: Cortex
Cx30: Connexin 30
DAB: 3,3’-Diaminobenzidine tetra-hydrochloride
DEGs: Differentially expressed genes
ERK: Extracellular signal-regulated kinase
FDR: False rate discovery
GFAP: Glial fibrillary acidic protein
GLAST: Glutamate transporter GLAST
GLT1: Glutamate transporter 1
HIPPO: Hippocampus
Iba1: Ionized calcium binding adapter molecule 1
IL-12: Interleukin 12
IL-15: Interleukin 15
IL-8: Interleukin 8
LDP/LTP: Long-term potentiation and long-term depression
mpi: Months post-last injury
HC: Healthy controls
HD: Huntington’s disease
MS: Multiple sclerosis
NDS: Normal donkey serum
NFκB: Nuclear factor kappa beta
PBS: Phosphate-buffered saline
PD: Parkinson’s disease
PSP: Progressive supranuclear palsy
r-mTBI: Repetitive mild traumatic brain injury
RT: Room temperature
STAT3: Signal transducer and activator of transcription 3
TBI: Traumatic brain injury
TBST: Tris-base saline Tween 20
WT: Wild type
