# Supplementary figures and images for: Overexpression of pathogenic tau in astrocytes causes a reduction in AQP4 and GLT1, an immunosuppressed phenotype and unique transcriptional responses to repetitive mild TBI without appreciable changes in tauopathy
Source: J Neuroinflammation. 2024 May 15;21:130. doi: 10.1186/s12974-024-03117-4 (PMC11096096; doi:10.1186/s12974-024-03117-4)

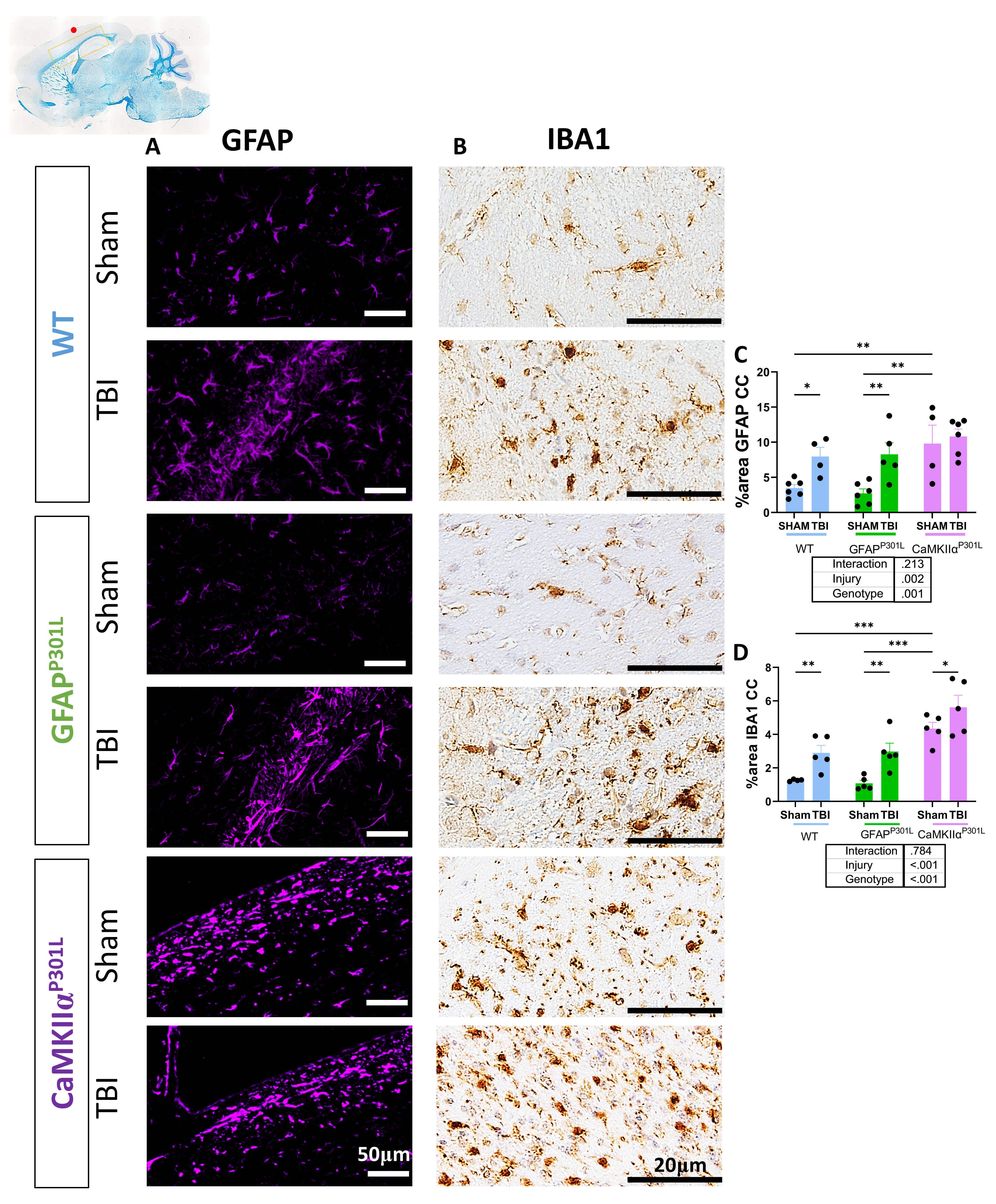

Supplement: Supplementary file 1 — Suuplemenatry Material 1: Figure 1: Astrocyte reactivity (GFAP) and microglial reactivity (Iba1) in the corpus callosum (CC) of WT, GFAPP301L and CaMKIIαP301L mice 3-months after r-mTBI/sham injury. Top right image is the overview of the region of interest (yellow box) where the images were collected from (red dot indicates the impact site). Qualitative images of GFAP and Iba1 in the CC (A and B, respectively) of WT mice (top-two panels), GFAPP301L mice (middle-two panels) and CaMKIIαP301L mice (bottom-two panels) 3-months after r-mTBI/sham injury. Images were captured at x20 magnification. Percentage area of GFAP (C) and Iba1 (D) in the CC (n=5-6 per group per genotype). Data were analyzed by Two-Way ANOVA followed by the Benjamini, Krieger, and Yekuteli test. Table under the graph details injury and genotype effects and their interaction after Two-way ANOVA. Asterisks denote: *P<0.05; **P<0.01 and ***P<0.001 for post-hoc analyses. [file 12974_2024_3117_MOESM1_ESM.png]

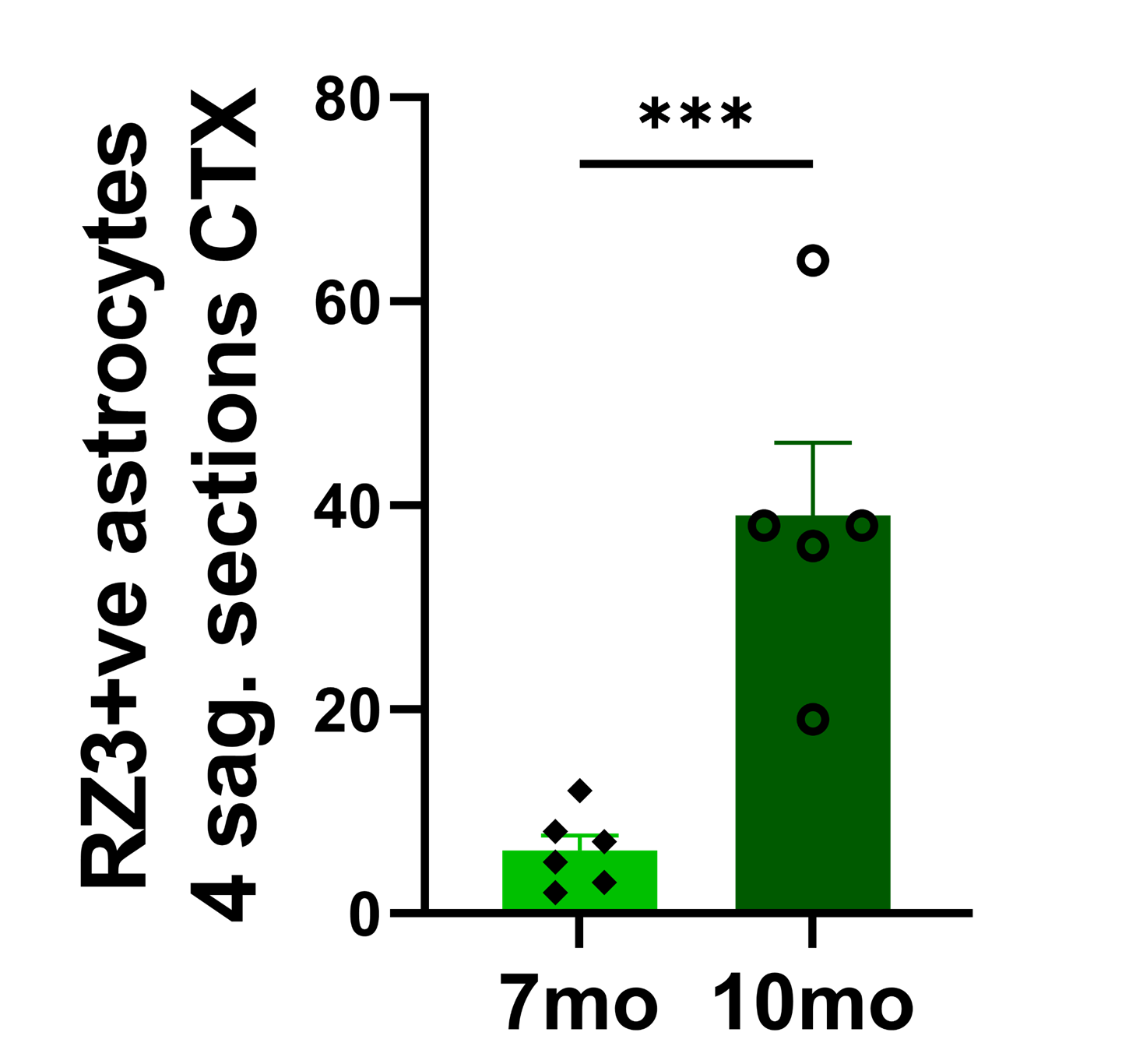

Supplement: Supplementary file 2 — Suuplementary Material 2: Figure 2: Tau astrogliopathy in the cortex of GFAPP301L mice at 7 and 10 months of age. RZ3/GFAP+ cells in the cortex from 4 serial sagittal sections at 7 and 10 months of age (n=5-6 per group). t-test analysis yielded significant changes p<0.001*** [file 12974_2024_3117_MOESM2_ESM.png]

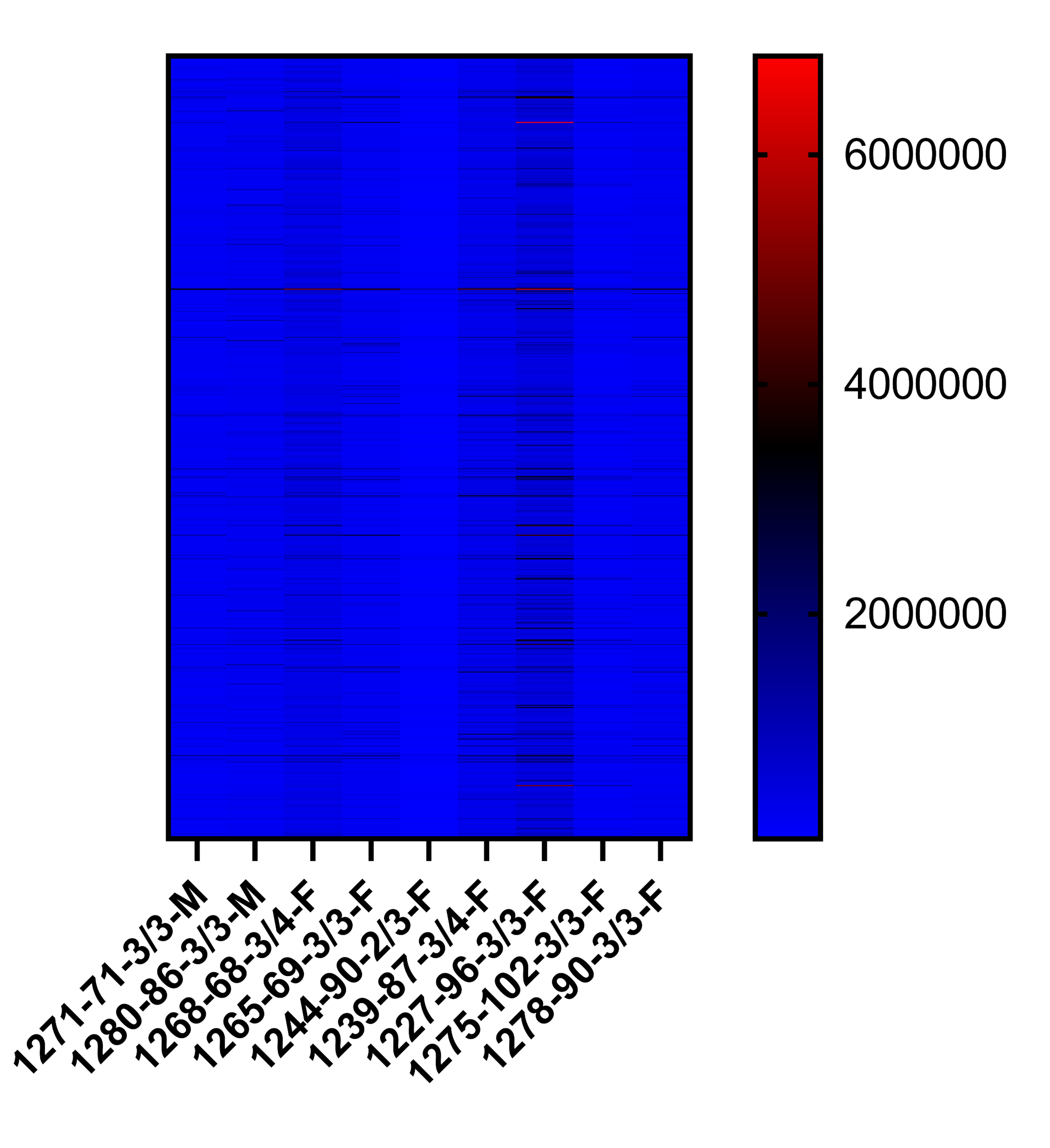

Supplement: Supplementary file 3 — Supplementary Material 3: Figure 3: Heat map revealing expression levels of all genes in the microarray of all healthy control (HC) cases and brief clinical demographics of CTE and HC cohorts. Heat map depicts relative intensity score (i.e., expression levels) of all genes in the microarray from all healthy control (HC) cases (n=9). Upregulated and downregulated genes are depicted in red and blue, respectively. [file 12974_2024_3117_MOESM3_ESM.png]
