# Supplementary material for: Overexpression of pathogenic tau in astrocytes causes a reduction in AQP4 and GLT1, an immunosuppressed phenotype and unique transcriptional responses to repetitive mild TBI without appreciable changes in tauopathy
Source: J Neuroinflammation. 2024 May 15;21:130. doi: 10.1186/s12974-024-03117-4 (PMC11096096; doi:10.1186/s12974-024-03117-4)
Supplement: Supplementary file 4 — Supplementary Material 4: Table 1. Antibodies used. Abbreviations: GFAP, Glial Fibrillary Acidic Protein; Iba1, Ionized calcium-binding adaptor molecule; AQP4, aquaporin 4; GLAST, Glutamate transporter; GLT1, Glutamate transporter 1. IF, Immunofluorescence; IHC, Immunohistochemistry; WB, Western blotting. [file 12974_2024_3117_MOESM4_ESM.docx]

**SUPPLEMENTARY TABLES**

| **Target protein** | **Dilution primary antibody** | **Host species** | **Vendor** | **Cat. number** | **Dilution secondary antibody** | **Protocol** |
| --- | --- | --- | --- | --- | --- | --- |
| AT8  (pTau Ser202/ Thr205) | 1:200 | Mouse | Invitrogen | MN1020 | 1:500  AlexaFluor 568 | IF |
| GFAP | 1:1000 | Chicken | Aves Labs | #GFAP | 1-500  AlexaFluor 647 |  |
| RZ3 (pThr231) | 1:200 | Mouse | Dr. Peter Davies |  | 1:500 AlexaFluor 568 |  |
| Iba1 | 1:1000 | Rabbit | Wako | 019-19741 | HRP-conjugated | IHC |
| AQP4 | 1:2000 | Rabbit | Millipore Sigma | HPA014784 | 1:2000 | WB |
| CP13 (ptau: Ser202) | 1:1000 | Mouse | Dr. Peter Davies |  | 1:2000 |  |
| DA9 (total tau) | 1:2000 | Mouse | Dr. Peter Davies |  | 1:2000 |  |
| GLAST | 1:1000 | Rabbit | Proteintech | 20785-1-AP | 1:2000 |  |
| GLT1 | 1:1000 | Mouse | LsBio | LS-B6724-50 | 1:2000 |  |
| PHF1 (ptau: Ser396/404) | 1:1000 | Mouse | Dr. Peter Davies |  | 1:2000 |  |
| RZ3 (ptau: Thr231) | 1:1000 | Mouse | Dr. Peter Davies |  | 1:2000 |  |

**Supplementary Table 1. Antibodies used.** Abbreviations: GFAP, Glial Fibrillary Acidic Protein; Iba1, Ionized calcium-binding adaptor molecule; AQP4, aquaporin 4; GLAST, Glutamate transporter; GLT1, Glutamate transporter 1; PSD95, Post-synaptic density 95. IF, Immunofluorescence; IHC, Immunohistochemistry; WB, Western blotting.
